# Inflammation-Induced Adverse Pregnancy and Neonatal Outcomes Can Be Improved by the Immunomodulatory Peptide Exendin-4

**DOI:** 10.3389/fimmu.2018.01291

**Published:** 2018-06-18

**Authors:** Valeria Garcia-Flores, Roberto Romero, Derek Miller, Yi Xu, Bogdan Done, Chharitha Veerapaneni, Yaozhu Leng, Marcia Arenas-Hernandez, Nabila Khan, Bogdan Panaitescu, Sonia S. Hassan, Luis Marat Alvarez-Salas, Nardhy Gomez-Lopez

**Affiliations:** ^1^Perinatology Research Branch, Program for Perinatal Research and Obstetrics, Division of Intramural Research, Eunice Kennedy Shriver National Institute of Child Health and Human Development, NICHD/NIH/DHHS, Detroit, MI, United States; ^2^Perinatology Research Branch, Program for Perinatal Research and Obstetrics, Division of Intramural Research, Eunice Kennedy Shriver National Institute of Child Health and Human Development, NICHD/NIH/DHHS, Bethesda, MD, United States; ^3^Department of Obstetrics and Gynecology, Wayne State University School of Medicine, Detroit, MI, United States; ^4^Departamento de Genética y Biología Molecular, Cinvestav, Mexico City, Mexico; ^5^Department of Obstetrics and Gynecology, University of Michigan, Ann Arbor, MI, United States; ^6^Department of Epidemiology and Biostatistics, Michigan State University, East Lansing, MI, United States; ^7^Center for Molecular Medicine and Genetics, Wayne State University, Detroit, MI, United States; ^8^Department of Immunology, Microbiology and Biochemistry, Wayne State University School of Medicine, Detroit, MI, United States; ^9^Departamento de Biomedicina Molecular, Cinvestav, Mexico City, Mexico

**Keywords:** amniotic fluid, clinical chorioamnionitis, fetal inflammatory response syndrome, intra-amniotic infection/inflammation, M2 macrophages, neutrophils, regulatory T cells, preterm labor and birth

## Abstract

Preterm birth is the leading cause of neonatal morbidity and mortality worldwide. Inflammation is causally linked to preterm birth; therefore, finding an intervention that dampens maternal and fetal inflammatory responses may provide a new strategy to prevent adverse pregnancy and neonatal outcomes. Using animal models of systemic maternal inflammation [intraperitoneal injection of lipopolysaccharide (LPS)] and fetal inflammation (intra-amniotic administration of LPS), we found that (1) systemic inflammation induced adverse pregnancy and neonatal outcomes by causing a severe maternal cytokine storm and a mild fetal cytokine response; (2) fetal inflammation induced adverse pregnancy and neonatal outcomes by causing a mild maternal cytokine response and a severe fetal cytokine storm; (3) exendin-4 (Ex4) treatment of dams with systemic inflammation or fetal inflammation improved adverse pregnancy outcomes by modestly reducing the rate of preterm birth; (4) Ex4 treatment of dams with systemic, but not local, inflammation considerably improved neonatal outcomes, and such neonates continued to thrive; (5) systemic inflammation facilitated the diffusion of Ex4 through the uterus and the maternal–fetal interface; (6) neonates born to Ex4-treated dams with systemic inflammation displayed a similar cytokine profile to healthy control neonates; and (7) treatment with Ex4 had immunomodulatory effects by inducing an M2 macrophage polarization and increasing anti-inflammatory neutrophils, as well as suppressing the expansion of CD8+ regulatory T cells, in neonates born to dams with systemic inflammation. Collectively, these results provide evidence that dampening maternal systemic inflammation through novel interventions, such as Ex4, can improve the quality of life for neonates born to women with this clinical condition.

## Introduction

Preterm birth is one of the most common, yet harmful, obstetrical syndromes (1) and is the leading cause of perinatal morbidity and mortality worldwide (2–4). Up to 70% of all preterm birth are preceded by spontaneous preterm labor (5, 6), a syndrome comprised of multiple pathological processes (1). While many putative causes are associated with spontaneous preterm labor, the only one that is causally linked to preterm birth is inflammation/infection (7, 8). Inflammation can be due to microorganisms (i.e., intra-amniotic infection) or danger signals derived from necrosis and cellular stress (i.e., sterile intra-amniotic inflammation) (9–14). Systemically, intra-amniotic infection can be manifested as clinical chorioamnionitis, which refers to the presence of maternal fever associated with clinical signs (foul-smelling discharge and uterine tenderness as well as maternal and fetal tachycardia) and laboratory abnormalities such as leukocytosis (15–18). Locally, intra-amniotic infection is characterized by an increased white blood cell count (19–22) and elevated concentrations of cytokines (23) and lipid mediators (e.g., prostaglandins) (24–31) in the amniotic cavity. This local inflammatory response can indicate a systemic activation of the fetal innate immune system, a phenomenon referred to as fetal inflammatory response syndrome (FIRS) (32, 33). Clinically, FIRS is defined by elevated cytokines in the fetal plasma, such as IL-6 (34), and by the presence of the fetus-related histopathological lesions funisitis and chorionic vasculitis (35–37). Fetuses with FIRS are often born to mothers with subclinical microbial invasion of the amniotic cavity (32). If the infection reaches the fetus, it may result in a systemic fetal infection that can progress toward multiple organ dysfunction, septic shock, and death (38). Finding a treatment for the prevention of inflammation-induced adverse pregnancy outcomes (39, 40), which can target both the maternal and fetal inflammatory responses, is critical.

Several substances with anti-inflammatory properties have been suggested as possible candidates for the prevention of inflammation-induced adverse pregnancy outcomes (41). *In vivo* studies using pregnant mice have shown that antibodies against cytokines (42) or their receptors (43), cytokine antagonists (44, 45), cytokine-suppressive anti-inflammatory drugs (46), COX-2 inhibitors (47), hormones such as progesterone (48, 49) and human chorionic gonadotropin (50), resveratrol (51), resolvins (52), PPARγ agonists (53–56), statins (57), and probiotics (58) are potential anti-inflammatory therapies for preterm birth prevention. Yet, further investigation is still required to determine the efficacy and safety of such treatments (41). Herein, we propose the use of a peptide, exendin-4 (Ex4), as an alternative approach for preventing inflammation-induced preterm labor and birth and adverse neonatal outcomes. In general, peptides are selective and efficacious signaling molecules that bind to a specific cell-surface receptor, which triggers intracellular effects (59). Because of their attractive pharmacological profile and intrinsic properties as well as their specificity, peptides represent an excellent alternative for the design of novel therapeutic approaches with potential safety, tolerability, and efficacy in humans (59).

Exendin-4 is a glucagon-like peptide-1 receptor (GLP1R) agonist, which is commonly used to treat diabetes mellitus type 2 (60). GLP1R is expressed in pancreatic beta cells and activation of this receptor stimulates the adenylyl cyclase pathway, which results in increased synthesis and release of insulin (61). In addition to the pancreas, GLP1R is expressed in several other organs including the intestine, lung, kidney, breast, and brain (62). The widespread distribution of this receptor in organs has resulted in multiple studies examining this receptor as a target for the treatment of various diseases. For example, Ex4 reduces liver damage (63) as well as inflammation and atherosclerosis (64). This peptide also has protective effects in renal injury (65) and post-myocardial infarction (66) by reducing inflammation. Moreover, Ex4 has potent immunomodulatory effects in both mice and humans as evidenced by the following demonstrations: Ex4 (a) prevents inflammation-induced migration of macrophages and their release of pro-inflammatory cytokines *in vitro* (67) and *in vivo* (64); (b) improves neutropenia and decreases the systemic levels of pro-inflammatory cytokines in a rat model of endotoxemia (68); (c) reduces the expression of iNOS and the production of reactive oxygen species, as well as the release of pro-inflammatory cytokines by *in vitro* M1-polarized human macrophages (69, 70); (d) attenuates the release of pro-inflammatory cytokines (e.g., TNFα, IL-1β, and IL-6) and chemokines (e.g., CCL5/RANTES and CXCL10/IP-10) by peripheral blood mononuclear cells from type 2 diabetic patients, which is likely mediated by the suppression of the p38 MAPK pathway (71); and (e) suppresses subcutaneous tumor growth in a dose-dependent manner by increasing Th1 pro-inflammatory responses (e.g., IFNγ and IL-1) and reducing CD4+ Foxp3+ regulatory T cells (Tregs) (72). Therefore, we propose that Ex4 could prevent adverse pregnancy and neonatal outcomes by exhibiting immunomodulatory effects and dampening maternal and fetal inflammation.

The aims of this study were (1) to evaluate the maternal and fetal cytokine responses in systemic and local models of inflammation-induced preterm birth and adverse neonatal outcomes; (2) to determine whether an anti-inflammatory peptide, Ex4, can dampen the inflammation to prevent adverse pregnancy and neonatal outcomes; (3) to localize Ex4 in the maternal and fetal tissues; and (4) to investigate the anti-inflammatory properties of Ex4 on the neonatal immune response by measuring the plasma cytokine response, inflammation-related gene expression, M1–M2 macrophage polarization, pro- and anti-inflammatory neutrophil phenotypes, and CD4+ and CD8+ Treg subsets.

## Materials and Methods

### Animals

C57BL/6 (B6) mice were purchased from The Jackson Laboratory in Bar Harbor, ME, USA, and bred in the animal care facility at the C.S. Mott Center for Human Growth and Development at Wayne State University, Detroit, MI, USA. All mice were housed under a circadian cycle (12 h light/12 h dark). Females 8–12 weeks old were mated with males of the same background and proven fertility. Female mice were checked daily between 8:00 a.m. and 9:00 a.m. for the appearance of a vaginal plug, which indicated 0.5 days post coitum (dpc). Females were then placed into new cages, and their weights were monitored daily. A gain of two or more grams by 12.5 dpc confirmed pregnancy. All procedures were approved by the Institutional Animal Care and Use Committee at Wayne State University (Protocol No. A-07-03-15).

### Animal Models of Preterm Birth/Fetal Inflammatory Response

Intra-amniotic administration of lipopolysaccharide (LPS) (73): pregnant B6 mice were anesthetized on 16.5 dpc by inhalation of 2–3% isoflurane (Aerrane, Baxter Healthcare Corporation, Deerfield, IL, USA) and 1–2 L/min of oxygen in an induction chamber. Anesthesia was maintained with a mixture of 1.5–2% isoflurane and 1.5–2 L/min of oxygen. Mice were positioned on a heating pad and stabilized with adhesive tape. Fur removal from the abdomen and thorax was achieved by applying Nair cream (Church & Dwight Co., Inc., Ewing, NJ, USA) to those areas. Body temperature was maintained in the range of 37 ± 1°C and detected with a rectal probe (VisualSonics, Toronto, ON, Canada), and respiratory and heart rates were monitored by electrodes embedded in the heating pad. An ultrasound probe was fixed and mobilized with a mechanical holder, and the transducer was slowly moved toward the abdomen. Ultrasound-guided intra-amniotic injection of LPS (*Escherichia coli* O111:B4; Sigma-Aldrich, St. Louis, MO, USA) at a concentration of 100 ng (*n* = 8) dissolved in 25 µL of sterile 1× phosphate-buffered saline (PBS; Fisher Scientific Bioreagents, Fair Lawn, NJ, USA) was performed in each amniotic sac using a 30-G needle (BD PrecisionGlide Needle, Becton Dickinson, Franklin Lakes, NJ, USA). Controls were injected with 25 µL of sterile 1× PBS (*n* = 7). The syringe was stabilized by a mechanical holder (VisualSonics Inc., Toronto, ON, Canada). Following the ultrasound, mice were placed under a heat lamp for recovery (defined as when the mouse resumes normal activity, such as walking and responding), which typically occurred 10–20 min after removal from anesthesia. After recovery, mice were video monitored.

Intraperitoneal administration of LPS (73): pregnant B6 mice were intraperitoneally injected on 16.5 dpc with 10 µg of LPS (*Escherichia coli* 055:B5; Sigma-Aldrich) (*n* = 10) in 200 µL of PBS using a 26-G needle. Controls were injected with 200 µL of sterile 1× PBS (*n* = 8). Mice were video monitored.

### Video Monitoring

Pregnancy parameters including the rates of preterm birth and pup mortality were recorded *via* video camera (Sony Corporation, Tokyo, Japan). Preterm birth was defined as delivery occurring before 18.0 dpc, and its rate was represented by the percentage of females delivering preterm among the total number of mice injected. The rate of pup mortality for each litter was defined as the proportion of delivered pups found dead among the total litter size. Neonatal survival was recorded 1 week postpartum.

### Serum and Tissue Collection From Dams

Pregnant B6 mice were intraperitoneally or intra-amniotically injected with either LPS or PBS on 16.5 dpc, as described previously. On 17.5 dpc, mice were euthanized, and peripheral blood was collected by cardiac puncture and placed into a 1.5 mL safe-lock Eppendorf tube (Fisher Scientific, Hanover Park, IL, USA). Serum (*n* = 10 each) was separated from the maternal peripheral blood and stored at −20°C until analysis. Animal dissection to obtain the fetal lung (*n* = 10–21 each) and amniotic fluid (*n* = 5–14 each) was performed. The amniotic fluid was also collected from each amniotic sac with a 26-G needle and placed into a 0.5 mL safe-lock Eppendorf tube (Fisher Scientific). Amniotic fluid samples were centrifuged at 1,300 × *g* for 10 min at 4°C and the supernatant was separated and stored at −20°C until analysis.

### Ex4 Treatment

Pregnant B6 mice were intraperitoneally injected with 30 µg/kg of Ex4 (Enzo Life Sciences, Ann Arbor, MI, USA) diluted in sterile 1× PBS 6 h after intraperitoneal (*n* = 10) or intra-amniotic (*n* = 8) administration of LPS. Control pregnant mice were intraperitoneally injected with 30 µg/kg of Ex4 (*n* = 5) on 16.5 dpc. Pregnant mice were also injected with LPS alone either intraperitoneally (10 µg/200 µL, *n* = 10) or intra-amniotically (10 ng/25 µL, *n* = 8) on 16.5 dpc, and control mice received an intraperitoneal (200 µL, *n* = 8) or intra-amniotic (25 µL, *n* = 7) injection of 1× PBS alone on 16.5 dpc. Lower doses of Ex4 were also tested (10 and 20 µg/kg); however, these did not have protective effects (data not shown).

### Fluorescent *In Vivo* Imaging to Detect Ex4

Pregnant B6 mice were injected intraperitoneally with 30 µg/kg of Ex4 (Fluorescein-TRP^25^-Exendin-4, FLEX) (cat # AS-63899, Anaspec Inc., Fremont, CA, USA) 6 h after the intraperitoneal administration of LPS (*n* = 3). Control mice were injected with LPS, FLEX, or 200 µL of PBS alone at 16.5 dpc (*n* = 3 each). One hour after the second injection, the uterus, placenta, decidua, fetal membranes, and fetus were collected to perform imaging using an IVIS Spectrum (Caliper Life Sciences, Hopkinton, MA, USA) in epifluorescence mode.

### Plasma and Tissue Collection From Neonates

Pregnant B6 mice were injected intraperitoneally with 30 µg/kg of Ex4 6 h after intraperitoneal administration of LPS (*n* = 3). Control pregnant mice were injected with 200 µL of 1× PBS or Ex4 alone (*n* = 3 each). Thriving neonates (*n* = 12–14 per group) were euthanized at 15 days of age and the brain, thymus, lung, spleen, liver, and small and large intestine were collected. Plasma was also separated from the neonatal peripheral blood and stored at −20°C until analysis. For RNA studies, the neonatal brain, lung, liver, and small intestine were placed into RNAlater Stabilization Solution (Invitrogen by Thermo Fisher Scientific, Baltics UAB, Lithuania) according to the manufacturer’s instructions. For leukocyte isolation, the neonatal thymus, lung, spleen, liver, and large intestine were utilized.

### Chemokine/Cytokine Concentrations

Maternal serum, neonatal plasma, and amniotic fluid samples were assessed for chemokine/cytokine concentrations. The ProcartaPlex Mouse Cytokine & Chemokine Panel 1A 36-plex (Invitrogen by Thermo Fisher Scientific, Vienna, Austria) was used to measure the concentrations of IFNα, IFNγ, IL-12p70, IL-1β, IL-2, TNFα, GM-CSF, IL-18, IL-17A, IL-22, IL-23, IL-27, IL-9, IL-15/IL-15R, IL-13, IL-4, IL-5, IL-6, IL-10, Eotaxin (CCL11), IL-28, IL-3, LIF, IL-1α, IL-31, GRO-α (CXCL1), MIP-1α (CCL3), IP-10 (CXCL10), MCP-1 (CCL2), MCP-3 (CCL7), MIP-1β (CCL4), MIP-2 (CXCL2), RANTES (CCL5), G-CSF, M-CSF, and ENA-78 (CXCL5) in the serum, plasma, and amniotic fluid samples, according to the manufacturer’s instructions. Plates were read using the Luminex 100 SystemFill (Luminex, Austin, TX, USA), and analyte concentrations were calculated with ProcartaPlex Analyst 1.0 Software from Affymetrix, San Diego, CA, USA. The sensitivities of the assays were 3.03 pg/mL (IFNα), 0.09 pg/mL (IFNγ), 0.21 pg/mL (IL-12p70), 0.14 pg/mL (IL-1β), 0.10 pg/mL (IL-2), 0.39 pg/mL (TNFα), 0.19 pg/mL (GM-CSF), 9.95 pg/mL (IL-18), 0.08 pg/mL (IL-17A), 0.24 pg/mL (IL-22), 2.21 pg/mL (IL-23), 0.34 pg/mL (IL-27), 0.28 pg/mL (IL-9), 0.42 pg/mL (IL-15/IL-15R), 0.16 pg/mL (IL-13), 0.03 pg/mL (IL-4), 0.32 pg/mL (IL-5), 0.21 pg/mL (IL-6), 0.69 pg/mL (IL-10), 0.01 pg/mL (Eotaxin), 20.31 pg/mL (IL-28), 0.11 pg/mL (IL-3), 0.28 pg/mL (LIF), 0.32 pg/mL (IL-1α), 0.45 pg/mL (IL-31), 0.05 pg/mL (GRO-α), 0.13 pg/mL (MIP-1α), 0.26 pg/mL (IP-10), 3.43 pg/mL (MCP-1), 0.15 pg/mL (MCP-3), 1.16 pg/mL (MIP-1β), 0.37 pg/mL (MIP-2), 0.35 pg/mL (RANTES), 0.19 pg/mL (G-CSF), 0.02 pg/mL (M-CSF), and 5.67 pg/mL (ENA-78). Inter-assay and intra-assay coefficients of variation were less than 10%.

### RNA Isolation, cDNA Synthesis, and Reverse Transcription Quantitative Polymerase Chain Reaction Analysis

Total RNA was isolated from fetal (17.5 dpc) and neonatal (15 days of age) tissues using QIAshredders, RNase-Free DNase Sets, and RNeasy Mini Kits (all from Qiagen, Hilden, Germany), according to the manufacturer’s instructions. RNA concentrations and purity were assessed with the NanoDrop 1000 spectrophotometer (Thermo Scientific, Wilmington, DE, USA), and RNA integrity was evaluated with the Bioanalyzer 2100 (Agilent Technologies, Wilmington, DE, USA). Complementary (c)DNA was synthesized using SuperScript III First-Strand Synthesis SuperMix (Invitrogen by Thermo Fisher Scientific, Carlsbad, CA, USA). Gene expression profiling was performed on the BioMark™ System for high-throughput RT-qPCR (Fluidigm, San Francisco, CA, USA) with the TaqMan^®^ gene expression assays (Applied Biosystems, Life Technologies Corporation, Foster City, CA, USA) listed in Table S1 in Supplementary Material.

### Leukocyte Isolation

The neonatal lung, liver, and large intestine were cut into small pieces using fine scissors and enzymatically digested with StemPro Cell Dissociation Reagent (Accutase, Life Technologies, Grand Island, NY, USA) for 10 min at 37°C. The spleen and thymus were gently dissociated using two glass slides to prepare a leukocyte suspension as previously described (74). Leukocyte suspensions were filtered using a 35 µm cell strainer (Falcon, Tamaulipas, Mexico) and washed with 1× PBS.

### Immunophenotyping

Leukocyte suspensions from the neonatal tissues were stained using LIVE/DEAD Fixable Blue Dead Cell Stain Kit (Life Technologies) prior to incubation with extracellular and intracellular mAbs. Leukocyte suspensions were centrifuged at 1,250 × *g* for 7 min at 4°C and cell pellets were incubated for 10 min with the CD16/CD32 mAb (FcgIII/II Receptor; BD Biosciences, San Jose, CA, USA) and subsequently incubated with specific extracellular and intracellular fluorochrome-conjugated anti-mouse mAbs (Table S2 in Supplementary Material) for 30 min. After extracellular staining, the cells were washed with fluorescence-activated cell sorting (FACS) buffer (bovine serum albumin 0.1%, sodium azide 0.05%, 1× PBS) to remove excess Ab. For immunophenotyping of macrophages and neutrophils, following the extracellular staining, the cells from the neonatal lung, liver, and large intestine were fixed and permeabilized using the BD Cytofix/Cytoperm fixation and permeabilization solution (BD Biosciences). For immunophenotyping of Tregs, following the extracellular staining, the cells from the neonatal thymus and spleen were fixed and permeabilized using the Foxp3/Transcription Factor Staining Buffer Set (eBioscience, San Diego, CA, USA) prior to intranuclear Foxp3 staining.

Leukocyte subsets were gated within the viability gate. Immunophenotyping included identification of (1) macrophages (CD11b+ F4/80+) and their M1/M2 phenotypes by the expression of IL-10 and iNOS; (2) neutrophils (CD11b+ Ly6G+) and their anti- and pro-inflammatory phenotypes by the expression of IL-10 and iNOS; and (3) CD4+ and CD8+ Tregs (CD3+ CD4+ CD25+ FoxP3+ and CD3+ CD8+ CD25+ FoxP3+ cells, respectively).

The total number of specific leukocytes was determined using Count Bright absolute counting beads (Molecular Probes, Eugene, OR, USA). As a control for cellular autofluorescence, unstained cells were also treated in this same manner. Stained and unstained cell suspensions were re-suspended in 0.5 mL of FACS buffer and acquired using an LSRFortessa flow cytometer and FACSDiva 8.0 software (BD Biosciences). Data were analyzed using FlowJo software version 10 (Tree Star, Ashland, OR, USA).

### Statistical Analysis

Observational mouse data were analyzed using IBM SPSS, version 19.0, and all other analysis was performed with GraphPad Prism version 5. For rates of preterm birth and pup mortality, the statistical significance of group comparisons was assessed using Mann–Whitney *U* test. For RT-qPCR arrays, –ΔCt values were determined using multiple reference genes (*Gusb, Hsp90ab1, Gapdh*, and *Actb*) averaged within each sample to determine gene expression levels. A heat map was created for the group mean expression matrix (gene × group mean), with individual gene expression level being standardized first. The heat map represents the *Z*-scores of the mean (−ΔCt) and the hierarchical clustering using correlation distance. For flow cytometry data, the statistical significance of group comparisons was assessed using Mann–Whitney *U* tests. A *p*-value <0.05 was considered significant.

## Results

### Models of Inflammation-Induced Preterm Birth and Adverse Neonatal Outcomes

**Figure 1:**
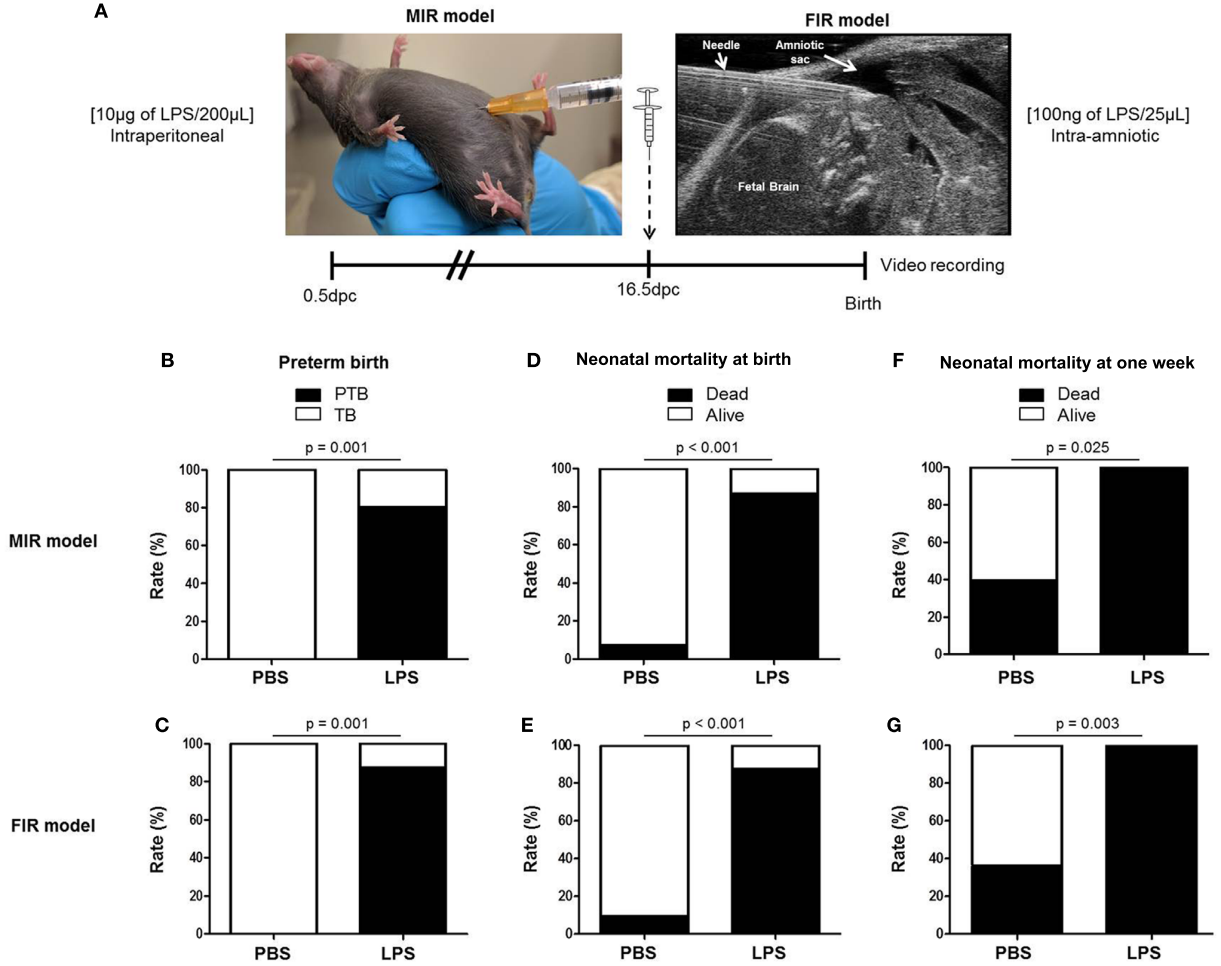
Models of inflammation-induced preterm birth and adverse neonatal outcomes. **(A)** On 16.5 days post coitum (dpc), pregnant mice were intraperitoneally (MIR model) (10 µg/200 µL) or intra-amniotically (FIR model) (100 ng/25 µL) injected with LPS or 1× PBS (200 or 25 µL) and mice were monitored until delivery. **(B,C)** Rate of preterm or term birth in the MIR and FIR models. **(D,E)** Rate of neonatal mortality at birth in the MIR and FIR models. **(F,G)** Rate of neonatal mortality at one week of age in the MIR and FIR models. *n* = 7–10 dams with litters per group. Abbreviations: PTB, preterm birth, TB, term birth; PBS, phosphate-buffered saline; LPS, lipopolysaccharide.

We first compared our two previously established models of inflammation-induced preterm birth: systemic administration of LPS *via* intraperitoneal injection (maternal inflammatory response model, MIR) and local administration of LPS *via* intra-amniotic injection (fetal inflammatory response model, FIR) (73). We injected mice intra-amniotically (100 ng/25 µL) or intraperitoneally (10 µg/200 µL) with LPS (or PBS controls) and observed pregnancy outcomes (Figure 1A). Both the MIR and FIR models resulted in a high rate of preterm birth (80 and 87.5%, respectively) while all of the controls injected with PBS delivered at term (Figures 1B,C). The rate of pup mortality at birth was greater than 85% in both the MIR and FIR models, which was significantly higher than that of controls (Figures 1D,E). At 1 week of age, no pups from dams that received either systemic or local administration of LPS survived (Figures 1F,G). These results demonstrate that a large LPS insult administered systemically or a lower dose given intra-amniotically induces adverse pregnancy and neonatal outcomes.

### The Maternal Cytokine Response in the MIR and FIR Models

**Figure 2:**
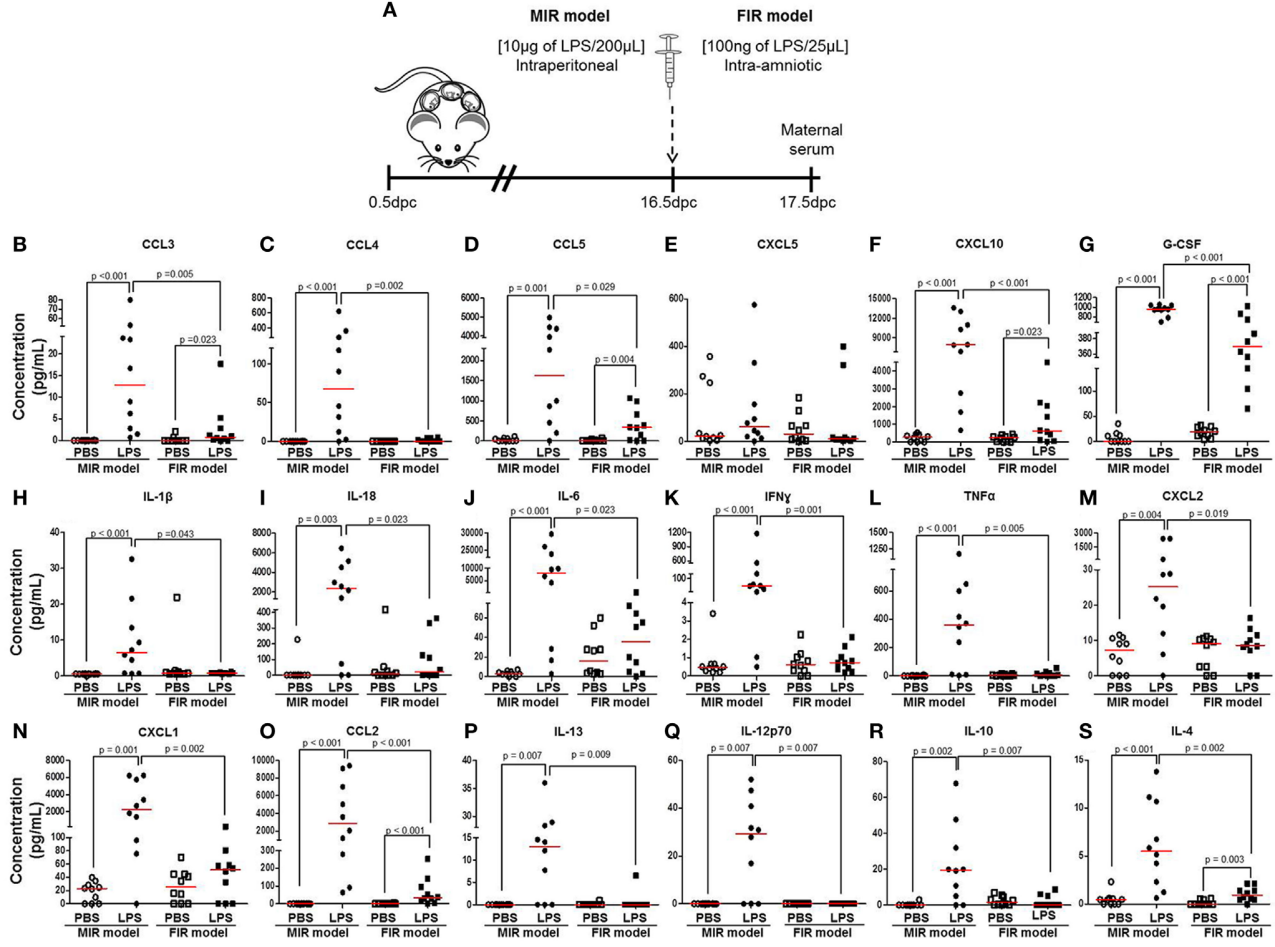
The maternal cytokine response in the MIR and FIR models. **(A)** On 16.5 days post coitum (dpc), pregnant mice were intraperitoneally (MIR model) (10 µg/200 µL) or intra-amniotically (FIR model) (100 ng/25 µL) injected with lipopolysaccharide (LPS) or 1× phosphate-buffered saline (PBS) (200 or 25 µL), and on 17.5 dpc maternal serum was collected for cytokine multiplex analysis. Concentrations of **(B)** CCL3, **(C)** CCL4, **(D)** CCL5, **(E)** CXCL5, **(F)** CXCL10, **(G)** G-CSF, **(H)** IL-1β, **(I)** IL-18, **(J)** IL-6, **(K)** IFNγ, **(L)** TNFα, **(M)** CXCL2, **(N)** CXCL1, **(O)** CCL2, **(P)** IL-13, **(Q)** IL-12p70, **(R)** IL-10, and **(S)** IL-4 in the maternal serum. *n* = 10 dams per group.

Next, we measured cytokine concentrations in the maternal circulation to evaluate the systemic inflammatory response in both the MIR and FIR models (Figure 2A). In the MIR model, there were significantly higher serum concentrations of 30 cytokines compared with PBS controls (Figures 2B–D,F–S; Figures S1A–D,F–J,L,N–P in Supplementary Material). In the FIR model, however, only seven cytokine concentrations were higher compared with controls (Figures 2B,D,F,G,O,S; Figure S1O in Supplementary Material). Interestingly, from the 34 cytokine concentrations reported herein, 25 of these were significantly higher in the MIR model than those in the FIR model (Figures 2B–D,F–S; Figures S1A,D,F,H–J,N–P in Supplementary Material). Therefore, the MIR model is characterized by a stronger maternal cytokine response than the FIR model.

### The Fetal Cytokine Response in the MIR and FIR Models

**Figure 3:**
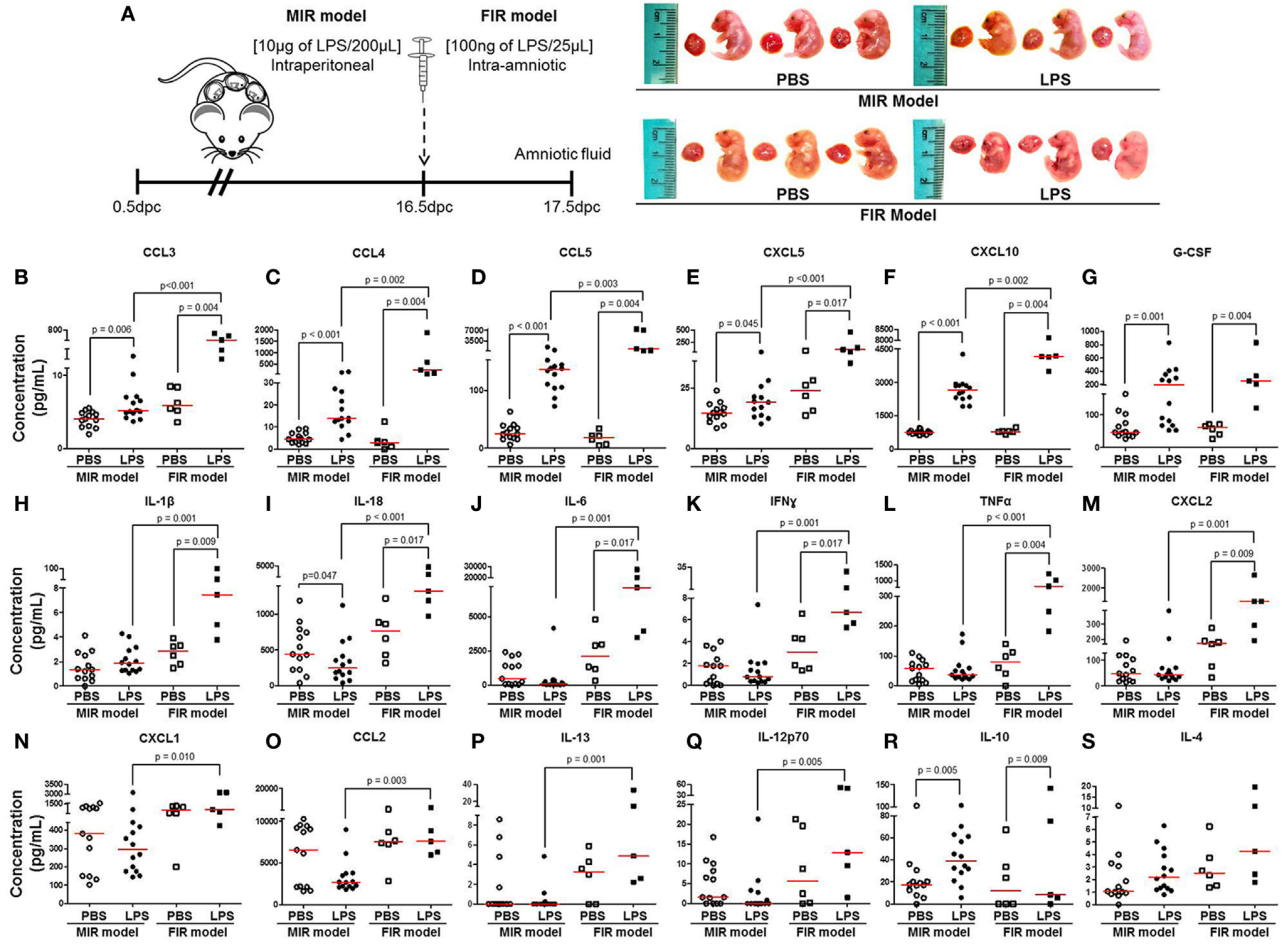
The fetal inflammatory response in the MIR and FIR models. **(A)** On 16.5 days post coitum (dpc), pregnant mice were intraperitoneally (MIR model) (10 µg/200 µL) or intra-amniotically (FIR model) (100 ng/25 µL) injected with lipopolysaccharide (LPS) or 1× phosphate-buffered saline (PBS) (200 or 25 µL), and on 17.5 dpc amniotic fluid was collected for cytokine multiplex analysis. Photographs of fetuses from dams with MIR or FIR are shown. Concentration of **(B)** CCL3, **(C)** CCL4, **(D)** CCL5, **(E)** CXCL5, **(F)** CXCL10, **(G)** G-CSF, **(H)** IL-1β, **(I)** IL-18, **(J)** IL-6, **(K)** IFNγ, **(L)** TNFα, **(M)** CXCL2, **(N)** CXCL1, **(O)** CCL2, **(P)** IL-13, **(Q)** IL-12p70, **(R)** IL-10, and **(S)** IL-4 in the amniotic fluid. *n* = 5–14 dams per group.

The fetal inflammatory response is associated with elevated IL-6 in the amniotic fluid (32, 33). Therefore, we collected amniotic fluid from the MIR and FIR models and measured cytokine concentrations (Figure 3A, left panel). No apparent differences were found between fetuses of dams injected with LPS intraperitoneally (MIR model) and their control counterparts; yet, fetuses of dams injected with LPS intra-amniotically (FIR model) seemed smaller and friable compared with those from PBS injected controls (Figure 3A, right panel). We found higher concentrations of 16 cytokines in the amniotic fluid in the MIR model when compared with its control (Figures 3B–G,R; Figures S2B,E,H–N in Supplementary Material). In the FIR model, we similarly detected an elevation in the amniotic fluid concentrations of 13 cytokines compared with its control (Figures 3B–M,R). However, the concentrations of 15 amniotic fluid cytokines were significantly higher in the FIR model than in the MIR model (Figures 3B–F,H–Q). Amniotic fluid concentrations of IL-4 were unchanged in the MIR and FIR models compared to their controls, and there was no significant difference when comparing the two models (Figure 3S). These results indicate that the fetal inflammatory response is more severe when the insult is given intra-amniotically than when given systemically.

### Inflammatory Gene Expression in the Fetal Lung

**Figure 4:**
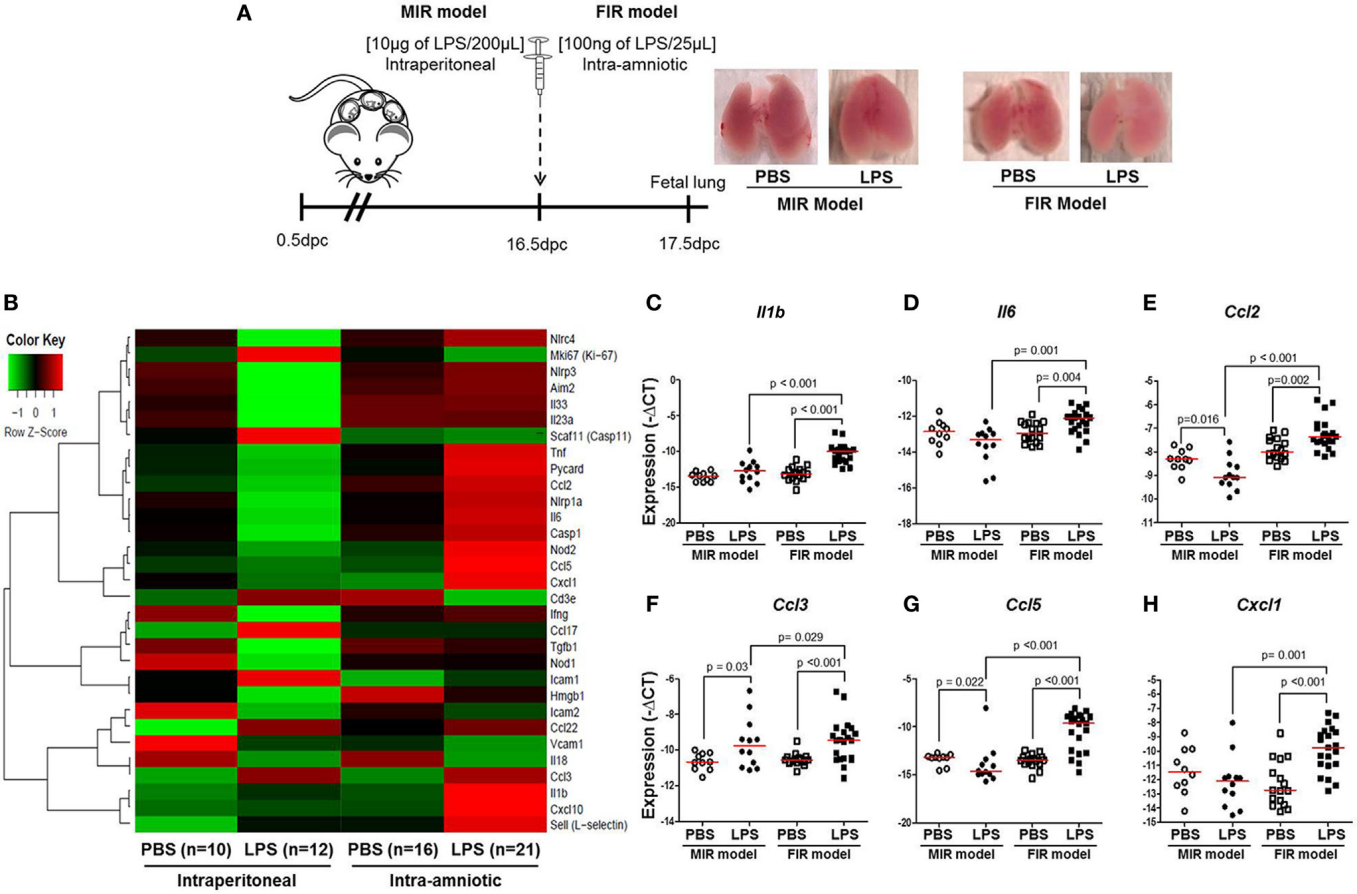
Inflammatory gene expression in the fetal lung. **(A)** On 16.5 days post coitum (dpc), pregnant mice were intraperitoneally (MIR model) (10 µg/200 µL) or intra-amniotically (FIR model) (100 ng/25 µL) injected with lipopolysaccharide (LPS) or 1× phosphate-buffered saline (PBS) (200 or 25 µL), and on 17.5 dpc fetal lung was collected for gene expression analysis. Photographs of fetal lungs from dams with MIR or FIR are shown. **(B)** Heat map visualization of gene expression in fetal lung tissue. Expression of **(C)** *Il1b*, **(D)** *Il6*, **(E)** *Ccl2*, **(F)** *Ccl3*, **(G)** *Ccl5*, and **(H)** *Cxcl1* in the fetal lung. *n* = 10–21 dams with litters per group.

Intra-amniotic inflammation is associated with fetal lung damage (75–77) and bronchopulmonary disorder (78–81). We then evaluated the expression of inflammation-associated genes in fetal lungs in both the MIR and FIR models (Figure 4A). No apparent differences were found between the lungs from fetuses of dams intraperitoneally injected with LPS (MIR model) compared with their controls; yet, the lungs from fetuses of dams with FIR seemed pallid compared with controls (Figure 4A). The heatmap array shown in Figure 4B indicated that, in the MIR model, there is a downregulation of inflammation-related genes in the fetal lungs, whereas in the FIR model, there is an upregulation of such genes (Figure 4B). When the expression of specific inflammatory genes in the fetal lung was plotted, we observed that *Il1b, Il6, Ccl2, Ccl3, Ccl5*, and *Cxcl1* were significantly upregulated in the FIR model compared with its control (Figures 4C–H). In the MIR model, however, only *Ccl3* was upregulated in the fetal lungs compared with its control (Figure 4F). Indeed, the expression of *Ccl2* and *Ccl5* was downregulated in the MIR model (Figures 4E,G). Together, these data demonstrate that intra-amniotic microbial products can cause an overexpression of inflammation-related genes in the fetal lungs, whereas maternal systemic inflammation seems to have the opposite effect.

### Treatment With Ex4 Improves Adverse Pregnancy and Neonatal Outcomes

**Figure 5:**
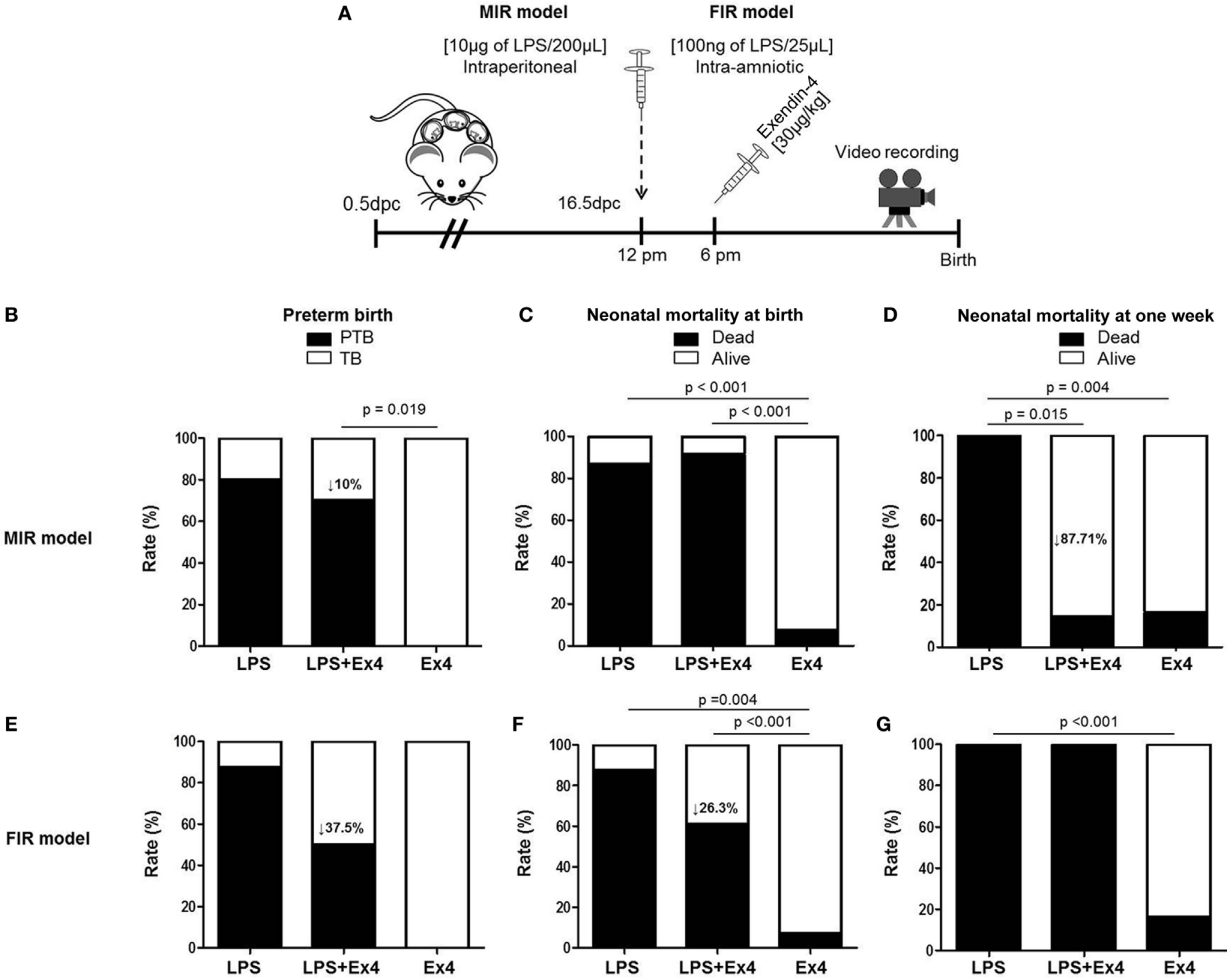
Treatment with Ex4 improves adverse pregnancy and neonatal outcomes. **(A)** On 16.5 days post coitum (dpc), pregnant mice were intraperitoneally (MIR model) (10 µg/200 µL) or intra-amniotically (FIR model) (100 ng/25 µL) injected with LPS and injected intraperitoneally with 30 µg/kg Ex4. Pregnant mice were also intraperitoneally (10 µg/200 µL) or intra-amniotically (10 ng/25 µL) injected with LPS or Ex4 alone on 16.5 dpc. Mice were monitored until delivery. **(B,E)** Rate of preterm birth in the MIR and FIR models. **(C,F)** Rate of neonatal mortality at birth in the MIR and FIR models. **(D,G)** Rate of neonatal mortality at one week of age in the MIR and FIR models. *n* = 5–10 dams with litters per group. Abbreviations: PTB, preterm birth, TB, term birth; Ex4, exendin-4; dpc, days post coitum; LPS, lipopolysaccharide.

In order to dampen the inflammation caused in the MIR and FIR models, we investigated whether an anti-inflammatory peptide, Ex4 (63–66, 69, 70), could reduce or prevent adverse pregnancy and neonatal outcomes (Figure 5A). Ex4 treatment caused a 10% reduction in the rate of preterm birth in the MIR model compared with dams that received only LPS (Figure 5B). Pups from the MIR model treated with Ex4 had a similar rate of mortality at birth compared with those without Ex4 treatment (Figure 5C). Interestingly, live-born pups from the MIR model which had received Ex4 treatment continued to thrive, whereas those born to dams without treatment died shortly after birth (Figure 5D).

In the FIR model, dams treated with Ex4 had a 37.5% decrease in the rate of preterm birth (Figure 5E). In addition, pups from the FIR model treated with Ex4 had a 26.3% decrease in mortality at birth compared with those born to untreated dams (Figure 5F); however, none of the pups from the FIR model survived to 1 week of age regardless of Ex4 treatment (Figure 5G).

In both the MIR and FIR models, treatment with Ex4 alone did not induce adverse pregnancy or neonatal outcomes (Figures 5B–G). Mice that received an intraperitoneal or intra-amniotic injection of 1× PBS did not present adverse pregnancy or neonatal outcomes (data not shown).

Collectively, these results show that Ex4 treatment can modestly reduce the rate of preterm delivery in dams with MIR or FIR. Importantly, Ex4 treatment can alleviate adverse neonatal outcomes in dams with systemic maternal inflammation but not in those with intra-amniotic inflammation.

### Ex4 Is Localized in the Uterus and Maternal–Fetal Interface

**Figure 6:**
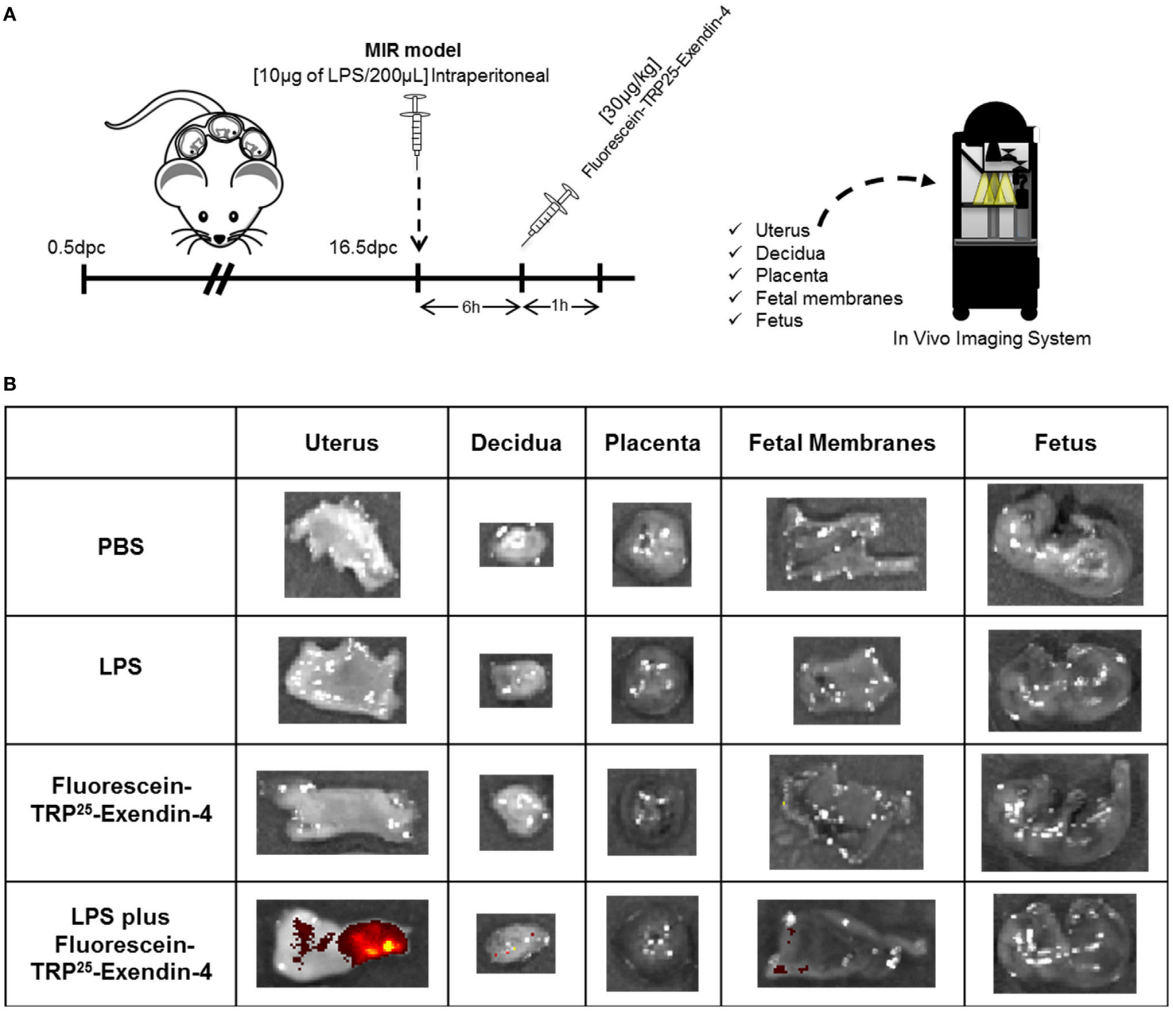
Exendin-4 is localized in the uterus and maternal–fetal interface. **(A)** On 16.5 days post coitum (dpc), pregnant mice were intraperitoneally injected with: (1) 1× phosphate-buffered saline (PBS) (200 µL); (2) lipopolysaccharide (LPS) (10 µg/200 µL); (3) Fluorescein-TRP^25^-Exendin-4 (FLEX) (30 µg/kg) alone; and (4) LPS followed by treatment with FLEX (30 µg/kg). Imaging was performed 1 h after the second injection. **(B)** Representative images taken with the *In Vivo* Imaging System showing the fluorescence of FLEX in the uterus, decidua, placenta, fetal membranes, and fetus. *n* = 3 per group.

Since treatment with Ex4 had beneficial effects in the MIR model, we next investigated the localization of this peptide in the maternal and fetal tissues using a fluorescence-labeled Ex4 (FLEX), which fluoresces after binding to the GLP-1 receptor (82) (Figure 6A). No signal was observed in the control tissues from mice injected with PBS or LPS alone (Figure 6B). Ex4 was strongly detected in the uterus from mice injected with LPS and FLEX (Figure 6B). A few traces of Ex4 were also detected in the decidua and fetal membranes from mice injected with LPS and FLEX (Figure 6B). However, Ex4 was not detected in any of the maternal or fetal tissues in mice injected with FLEX alone (Figure 6B). These findings suggest that systemic inflammation facilitates the diffusion of Ex4 through the uterus and the maternal–fetal interface.

### Neonates Born to Dams With Systemic Inflammation and Treated With Ex4 Display a Similar Cytokine Profile to Healthy Neonates

**Figure 7:**
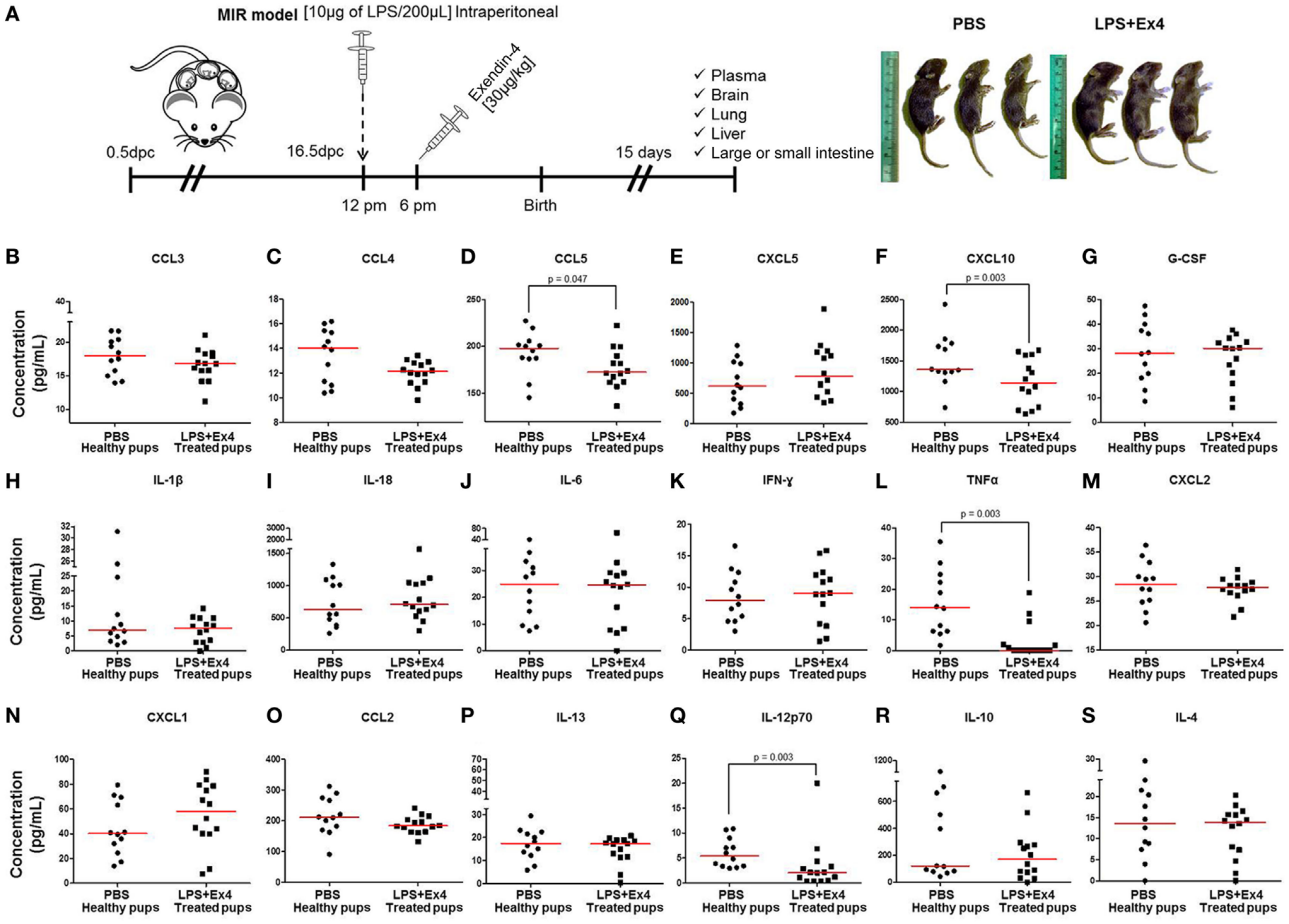
The cytokine profile of neonates born to dams with MIR and treated with exendin-4 (Ex4). **(A)** On 16.5 days post coitum (dpc), pregnant mice were intraperitoneally (10 µg/200 µL) injected with lipopolysaccharide (LPS) followed by treatment with Ex4 (30 µg/kg). Controls were injected with 1× phosphate-buffered saline (PBS, 200 µL) alone. At 15 days of age, neonatal plasma was collected for cytokine multiplex analysis. Concentrations of **(B)** CCL3, **(C)** CCL4, **(D)** CCL5, **(E)** CXCL5, **(F)** CXCL10, **(G)** G-CSF, **(H)** IL-1β, **(I)** IL-18, **(J)** IL-6, **(K)** IFNγ, **(L)** TNFα, **(M)** CXCL2, **(N)** CXCL1, **(O)** CCL2, **(P)** IL-13, **(Q)** IL-12p70, **(R)** IL-10, and **(S)** IL-4 in the neonatal plasma. *n* = 12–14 neonates per group.

Neonates born to dams with MIR and treated with Ex4 were indistinguishable from neonates born to control dams injected with Ex4 (data not shown) or PBS alone (Figure 7A, right panel). However, whether the immune system of these thriving pups was comparable to healthy neonates was unknown. We therefore compared the cytokine and cellular immune responses between neonates born to Ex4-treated dams with MIR and those from control dams. First, the plasma cytokine profile of 15-day-old neonates was determined (Figure 7A, left panel). Neonates born to dams with MIR which received Ex4 treatment had comparable plasma cytokine concentrations (31 of 35 cytokines) to healthy neonates (pups born to dams injected with PBS alone) (Figures 7B,C,E,G–K,M–P,R,S; Figures S3A–Q in Supplementary Material). Indeed, the plasma cytokine concentrations of CCL5, CXCL10, TNFα, and IL-12p70 were decreased in neonates born to dams with MIR which received Ex4 treatment (Figures 7D,F,L,Q) compared with healthy pups. Healthy pups treated with Ex4 alone had comparable, or even lower, plasma cytokine concentrations to healthy pups from dams injected with PBS alone (Figures S4A–R in Supplementary Material).

**Figure 8:**
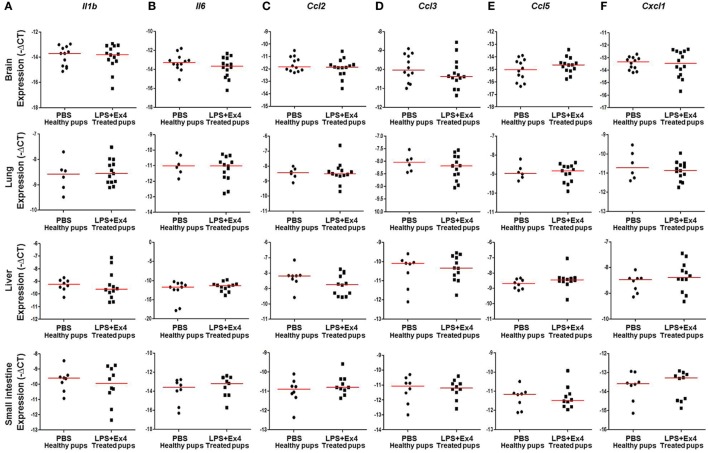
Inflammatory gene expression in neonates born to dams with MIR and treated with exendin-4 (Ex4). On 16.5 days post coitum (dpc), pregnant mice were intraperitoneally (10 µg/200 µL) injected with lipopolysaccharide (LPS) followed by treatment with Ex4 (30 µg/kg). Controls were injected with 1× phosphate-buffered saline (PBS, 200 µL) alone. At 15 days of age the neonatal brain, lung, liver, and small intestine were collected for gene expression analysis. Expression of **(A)** *Il1b*, **(B)** *Il6*, **(C)** *Ccl2*, **(D)** *Ccl3*, **(E)** *Ccl5*, and **(F)** *Cxcl1* in the neonatal brain, lung, liver and small intestine. *n* = 12–14 neonates per group.

Next, we determined the expression of inflammation-related genes in the neonatal brain, lung, liver, and small intestine (Figure 7A). The expression of *Il1b, Il6, Ccl2, Ccl3, Ccl5*, and *Cxcl1* in the brain, lung, liver, and small intestine from pups born to MIR dams which received Ex4 treatment was comparable to that of healthy pups (Figures 8A–F). No differences in the expression of such genes were observed between healthy pups born to dams injected with PBS (controls) or Ex4 alone (Figures S5A–X in Supplementary Material).

Collectively, these data show that Ex4 has anti-inflammatory properties in dams with MIR, which results in thriving and healthy neonates.

### Ex4 Treatment Induces an M1 → M2 Macrophage Polarization in the Neonate

**Figure 9:**
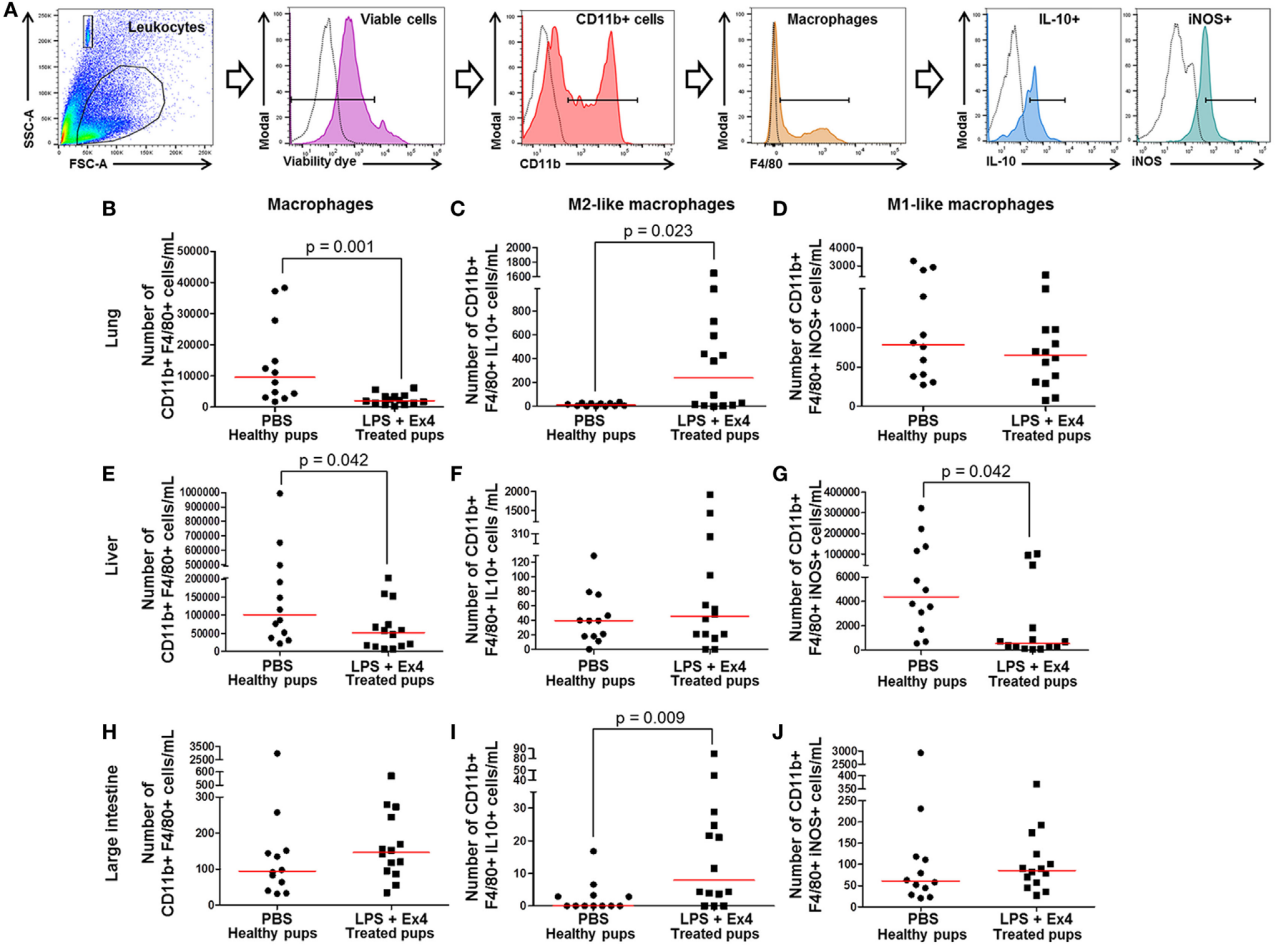
Exendin-4 (Ex4) treatment induces an M1 → M2 macrophage polarization in neonates. On 16.5 days post coitum (dpc), pregnant mice were intraperitoneally (10 µg/200 µL) injected with lipopolysaccharide (LPS) followed by treatment with Ex4 (30 µg/kg). Controls were injected with 1× phosphate-buffered saline (PBS, 200 µL) alone. At 15 days of age, the neonatal lung, liver, and large intestine were collected for immunophenotyping. **(A)** Gating strategy for M1- and M2-like macrophages. Dead cells were excluded using a viability dye. Empty histograms represent the autofluorescence control and colored histograms represent antibody fluorescent signals. Numbers of macrophages in the neonatal lung **(B)**, liver **(E)**, and large intestine **(H)**. Numbers of M2-like macrophages in the neonatal lung **(C)**, liver **(F)**, and large intestine **(I)**. Numbers of M1-like macrophages in the neonatal lung **(D)**, liver **(G)**, and large intestine **(J)**. *n* = 12–14 neonates per group.

The innate immune system has a central role in fetal and neonatal life (83, 84); therefore, we investigated whether neonates born to dams with MIR and treated with Ex4 had effects on lung, liver, and large intestine M1/M2 macrophage phenotypes. Macrophage immunophenotyping was performed in neonatal tissues (Figure 9A). The numbers of macrophages in the neonatal lung and liver were significantly reduced in neonates born to MIR dams which received Ex4 treatment when compared with those from controls (Figures 9B,E). The number of macrophages in the large intestine from neonates born to dams with MIR and treated with Ex4 was comparable to that of healthy pups (Figure 9H).

Next, M1-like (CD11b+ F4/80+ iNOS+) and M2-like (CD11b+ F4/80+ IL-10+) macrophages were immunophenotyped in these tissues (Figure 9A), as previously reported (49, 50, 54, 55). There was an increase in the number of M2-like macrophages in the lung and large intestine of neonates born to MIR dams with Ex4 treatment compared with controls (Figures 9C,I). The number of M2-like macrophages in the liver from neonates born to dams with MIR and treated with Ex4 was comparable to that of healthy pups (Figure 9F). Conversely, there was a decrease in the number of M1-like macrophages in the liver of neonates born to MIR dams with Ex4 treatment (Figure 9G). The number of M1-like macrophages in the lung and large intestine from neonates born to dams with MIR and treated with Ex4 was comparable to that of healthy pups (Figures 9D,J). Treatment of dams with Ex4 alone caused an M2 macrophage polarization in the neonatal tissues (Figures S6A–I in Supplementary Material). Taken together, these results indicate that Ex4 treatment of dams with MIR decreases the overall number of macrophages in the neonatal tissues, while promoting an M1 → M2 macrophage polarization.

### Ex4 Treatment Induces a Neutrophil Polarization in the Neonate

**Figure 10:**
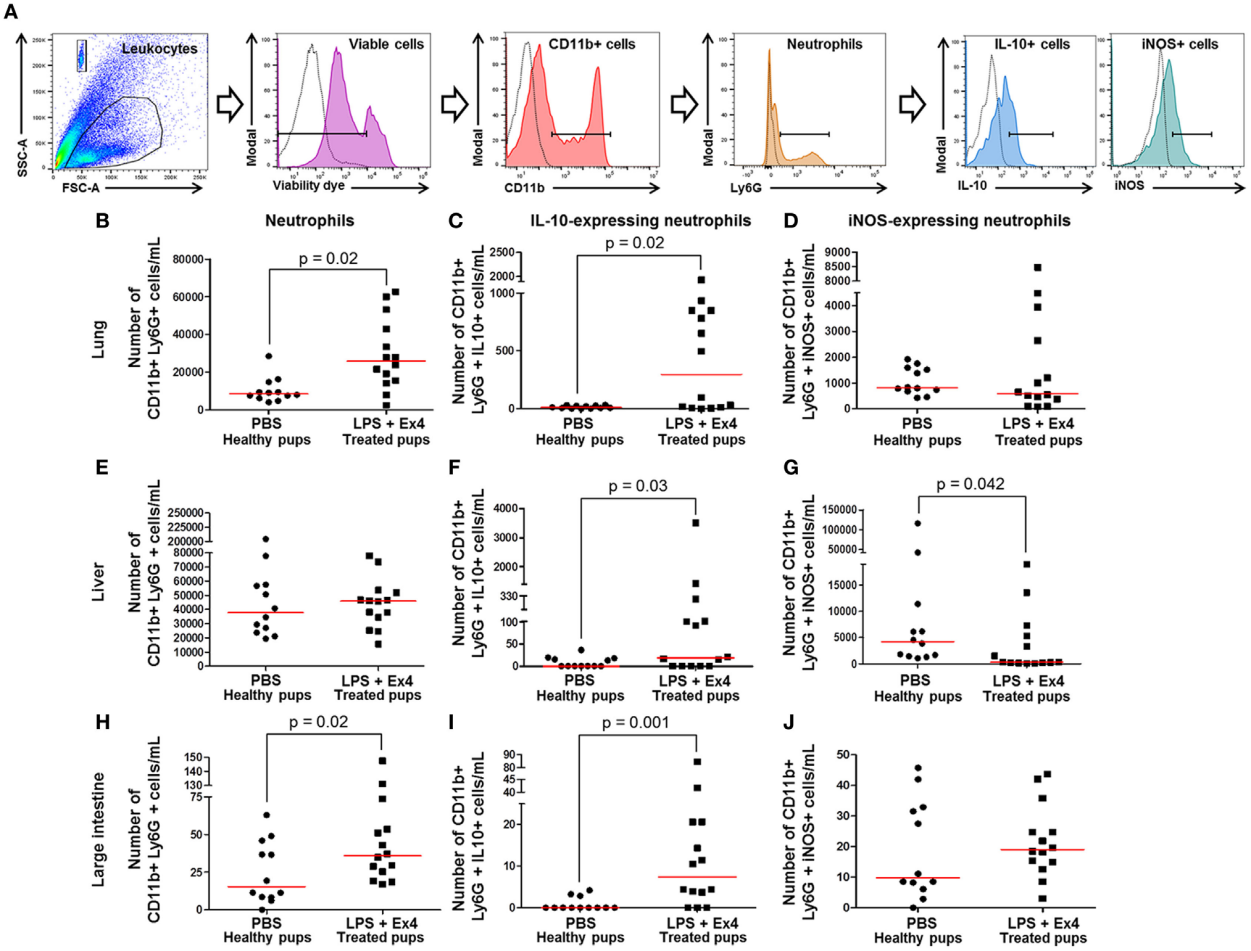
Exendin-4 (Ex4) treatment induces an increase in anti-inflammatory neutrophils in neonates. On 16.5 days post coitum (dpc), pregnant mice were intraperitoneally (10 µg/200 µL) injected with lipopolysaccharide (LPS) followed by treatment with Ex4 (30 µg/kg). Controls were injected with 1× phosphate-buffered saline (PBS, 200 µL) alone. At 15 days of age, the neonatal lung, liver, and large intestine were collected for immunophenotyping. **(A)** Gating strategy for neutrophil polarization. Dead cells were excluded using a viability dye. Empty histograms represent the autofluorescence control and colored histograms represent antibody fluorescent signals. Numbers of neutrophils in the neonatal lung **(B)**, liver **(E)**, and large intestine **(H)**. Numbers of IL-10-expressing neutrophils in the neonatal lung **(C)**, liver **(F)**, and large intestine **(I)**. Numbers of iNOS-expressing neutrophils in the neonatal lung **(D)**, liver **(G)**, and large intestine **(J)**. *n* = 12–14 neonates per group.

We next determined the total numbers of neutrophils (CD11b+ Ly6G+) as well as their expression of pro-inflammatory (iNOS) and anti-inflammatory (IL-10) cytokines in the neonatal lung, liver, and large intestine (Figure 10A). The total numbers of neonatal neutrophils were increased in the lung and large intestine of neonates born to MIR dams with Ex4 treatment when compared with PBS controls (Figures 10B,H). The number of neutrophils in the liver from neonates born to dams with MIR and treated with Ex4 was comparable to that of healthy pups (Figure 10E).

Neutrophil immunophenotyping in neonatal tissues was also performed (Figure 10A). There was an increase in the number of IL-10-expressing neutrophils in the lung, liver, and large intestine of neonates born to MIR dams with Ex4 treatment compared with controls (Figures 10C,F,I). By contrast, there was a decrease in the number of iNOS-expressing neutrophils in the liver of neonates born to MIR dams with Ex4 treatment compared with healthy pups (Figure 10G). The number of iNOS-expressing neutrophils in the lung and large intestine from neonates born to dams with MIR and treated with Ex4 was comparable to that of healthy pups (Figures 10D,J). Treatment of dams with Ex4 alone only caused an increase of pro- and anti-inflammatory neutrophils in the neonatal large intestine (Figures S7G–I in Supplementary Material). These results show that Ex4 treatment of dams with MIR increases the number of anti-inflammatory neutrophils in the neonatal tissues.

### Ex4 Treatment Reduces Neonatal CD8+ Tregs

**Figure 11:**
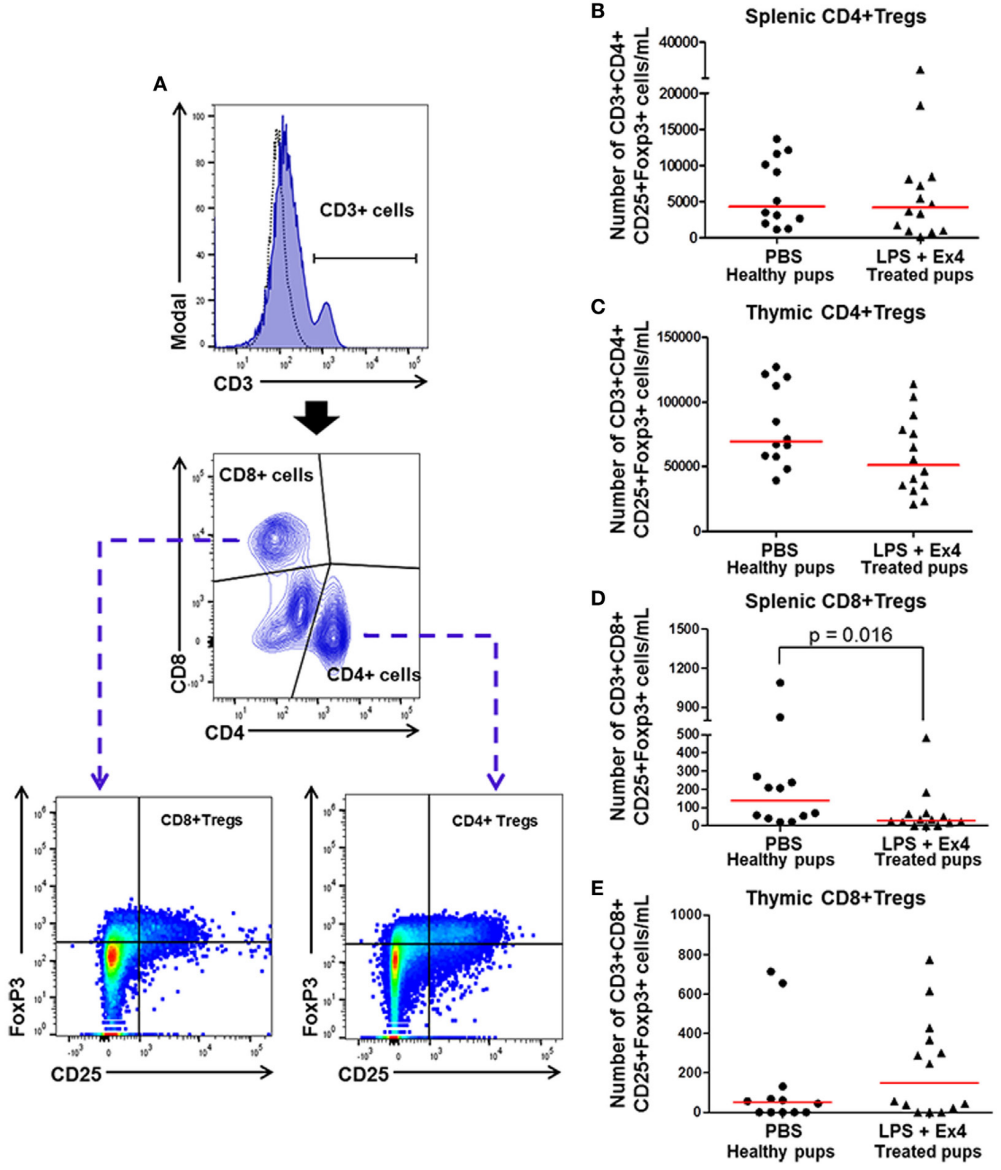
Exendin-4 (Ex4) treatment reduces neonatal CD8+ regulatory T cells (Tregs). On 16.5 days post coitum (dpc), pregnant mice were intraperitoneally (10 µg/200 µL) injected with lipopolysaccharide (LPS) followed by treatment with Ex4 (30 µg/kg). Controls were injected with 1× phosphate-buffered saline (PBS, 200 µL) alone. At 15 days of age, the neonatal spleen and thymus were collected for immunophenotyping. **(A)** Gating strategy for CD4+ and CD8+ T regulatory cells. Dead cells were excluded using a viability dye. Dotted histograms represent the autofluorescence control and colored histograms represent antibody fluorescent signals. CD4+ and CD8+ Tregs co-expressed CD25 and FoxP3. **(B,C)** Number of splenic and thymic CD4+ Tregs. **(D,E)** Number of splenic and thymic CD8+ Tregs. *n* = 12–14 neonates per group.

Regulatory T cells (Tregs) play a central role in both the developing fetus and in the neonate (83, 85–89). We then determined whether Ex4 treatment of dams with MIR is altering neonatal Tregs subsets (CD3+ CD4+ CD25+ FoxP3+ and CD3+ CD8+ CD25+ FoxP3+ cells) in the neonatal spleen and thymus (Figure 11A). No differences were observed in the number of splenic and thymic CD4+ Tregs between neonates born to Ex4 treated dams with MIR and healthy neonates (Figures 11B,C). The number of CD8+ Tregs was reduced in the spleen of neonates born to MIR dams with Ex4 treatment when compared with healthy neonates (Figure 11D); however, no differences were observed in thymic CD8+ Tregs (Figure 11E). No differences in the number of CD4+ and CD8+ Tregs were observed between neonates born to dams treated with Ex4 alone and those from PBS controls (Figures S8A–D in Supplementary Material). These results indicate that Ex4 may reduce neonatal inflammation by inhibiting the expansion of splenic CD8+ Tregs, which may have pro-inflammatory properties (90).

## Discussion

### Maternal and Fetal Inflammatory Responses in Preterm Labor

The intra-amniotic administration of a microbial product derived from Gram-negative bacteria induced preterm birth and neonatal death, as previously reported (73). This model is similar to the subclinical syndrome of preterm birth since (a) a low dose of LPS was injected, simulating the amniotic fluid concentrations of endotoxin found in women with spontaneous preterm labor (91); and (b) the intra-amniotic injection of low doses of LPS did not cause hypothermia, which is consistent with the fact that most of the intra-amniotic infections in women with spontaneous preterm labor occur in the absence of a temperature change (92, 93). Intra-amniotic infection is commonly associated with invasion of genital mycoplasmas, Gram-negative, and Gram-positive bacteria (15, 94–97) into the amniotic cavity. This infection can result in a maternal and/or fetal inflammatory response (15–17, 32, 33, 38, 98–103). This is consistent with the findings reported herein, in which we observe that the intra-amniotic administration of a microbial product results in both a maternal and fetal inflammatory response.

The systemic administration of a microbial product induced a severe maternal cytokine response but a mild fetal cytokine response, which caused preterm birth and neonatal death. A systemic maternal inflammatory response is observed in women with clinical chorioamnionitis (18) and acute pyelonephritis (104, 105), both clinical conditions associated with preterm birth (106–109) and adverse neonatal outcomes (107, 108). However, clinical chorioamnionitis results from intra-amniotic infection (15–18, 98), a condition which was not present in our model. On the other hand, acute pyelonephritis occurs independently of intra-amniotic infection and is not associated with a fetal inflammatory response (110), which resembles our MIR model.

### The Anti-Inflammatory Peptide Ex4 Rescues Inflammation-Induced Adverse Pregnancy and Neonatal Outcomes

The adverse pregnancy and neonatal outcomes observed in the MIR model were ameliorated by treatment with Ex4. This is consistent with a previous report showing that a GLP-1 analog, such as Ex4, dampened inflammatory pathways in a rat model of sepsis (111). GLP-1 receptors are present in the maternal (112) and fetal tissues (112–114). Herein, we found that Ex4 was mainly localized in the uterus and to a lesser extent in the decidua. These findings suggest that treatment with Ex4 has anti-inflammatory effects in the MIR model by primarily targeting the maternal tissues. This scenario explains why treatment with Ex4 did not rescue the adverse neonatal outcomes in the FIR model. Treatment with Ex4, however, did reduce the rate of preterm birth and neonatal mortality at birth in the FIR model, suggesting that a combined approach that targets both the maternal and fetal tissues may be the optimal strategy.

Neonates born to dams with systemic inflammation and treated with Ex4 thrived and displayed plasma and tissue cytokine profiles comparable to healthy neonates. Previous studies have shown that the GLP-1 receptor is expressed in the fetal tissues (112, 114), including the placenta (113). In addition, the administration of its agonists (Ex4 and liraglutide) increased the expression of surfactant protein A and B in the lung and amniotic fluid, which demonstrates the importance of the GLP-1 system in fetal development (113, 114). In the current study, we found that Ex4 was modestly detected in the fetal membranes, suggesting that this peptide could have partial effects on the tissues surrounding the fetus, which translated into thriving neonates.

### Innate and Adaptive Immune Responses in Thriving Neonates

Neonates born to dams with MIR and treated with Ex4 were indistinguishable from healthy control neonates. We, therefore, investigated whether the immune system of these thriving pups was comparable to healthy neonates.

Treatment with Ex4 induced an M2 macrophage polarization in neonates born to dams with systemic inflammation. This is consistent with previous studies demonstrating that glucagon-like peptides, such as Ex4, induce an M2 macrophage polarization *in vitro* (115) and *in vivo* (116). M2 macrophages are considered alternatively activated (117–121) and display anti-inflammatory properties through the production of IL-10 and upregulation of arginase-1 (119, 122–128). In addition, decidual M2 macrophages participate in maternal–fetal tolerance throughout pregnancy (55, 129–136), suggesting that Ex4 may also have effects at the maternal-fetal interface. Further studies are required to investigate the effects of GLP-1 analogs in the reproductive tissues and maternal–fetal interface.

Although adult neutrophils are a major component of the innate immune system, neonatal neutrophils tend to have quantitative and qualitative defects (84). For example, neonatal neutrophils have impaired chemotaxis, rolling adhesion, transmigration, and lamellipodia formation (137). Such innate immune cells also display impairments in anti-microbial mechanisms and are reduced in newborns presenting bacterial sepsis (137). In this study, we found that treatment with Ex4 caused an increase in anti-inflammatory neutrophils in neonates born to dams with systemic inflammation. These findings are in line with a previous report demonstrating that Ex4 can modulate neutropenia and dampen pro-inflammatory cytokines (68). Together, these results indicate that Ex4 treatment of dams with systemic inflammation modulates the fetal inflammatory response, which resulted in thriving neonates with increased anti-inflammatory neutrophils.

CD4+ Tregs play a central role in the immune response by preventing autoimmunity (inhibiting self-immune responses) and suppressing defensive immune responses to prevent host tissue damage (138–142). In the fetus, CD4+ Tregs are generated during pregnancy to participate in self-tolerance and tolerance to non-inherited antigens on chimeric maternal cells (85, 86). CD4+ Tregs are also implicated in the development of neonatal tolerance, where they suppress the development of donor-specific CD8+ T cell responses (83, 143). The fact that neonates born to dams with systemic inflammation and treated with Ex4 had normal numbers of CD4+ Tregs, which were comparable to those of healthy neonates, provides evidence that this peptide does not have deleterious effects on neonatal CD4+ Treg homeostasis.

CD8+ CD25+ T cells expressing FoxP3 seem to share phenotypic, functional, and mechanistic actions with classical CD4+ Tregs (144–146) and therefore are termed CD8+ Tregs. In neonates, CD8+ Tregs modulate Th2-cell-mediated pathology and autoimmunity (147, 148), suggesting that such cells shape the development of the immune system (83). In late pregnancy, however, maternal/decidual CD8+ CD25+ FoxP3+ T cells seem to have pro-inflammatory functions (90, 149). Herein, we found that treatment with Ex4 suppressed the expansion of CD8+ Tregs in neonates born to dams with systemic inflammation. The fact that Ex4 reduces CD8+ Tregs in the spleen of neonates born to dams with systemic inflammation suggests that such cells have pro-inflammatory rather than immunosuppressive functions. Yet, a functional assessment of neonatal CD8+ Tregs in the context of infection requires further investigation.

### Why Choose Ex4 for the Treatment of Inflammation-Induced Adverse Pregnancy Outcomes?

Several substances with anti-inflammatory properties have been suggested as possible candidates for the prevention of inflammation-induced adverse pregnancy outcomes; however, further investigation is still required to determine the efficacy and safety of such treatments (41). Herein, we provide data supporting the use of a peptide, Ex4, for the prevention of inflammation-induced preterm labor and birth and adverse neonatal outcomes. Importantly, we found that pregnant dams treated with Ex4 alone did not present adverse pregnancy and neonatal outcomes. Recent reports recognize the use of peptides as highly selective and efficacious therapeutic approaches since these are natural and are therefore relatively safe and well tolerated (59). Indeed, more than 60 peptide drugs have reached the market and approximately 140 peptide therapeutics are currently undergoing evaluation in clinical trials (59). In addition, a case report showed that the administration of Exenatide, the synthetic version of Ex4, to a pregnant woman during the first trimester was not associated with congenital malformation or other adverse pregnancy outcomes (150). Together, these findings suggest that peptides, such as Ex4, are well tolerated and safe for the mother and fetus/neonate. Yet, further research in larger animals is required to evaluate the safety of Ex4.

## Conclusion

The findings presented herein provide evidence that Ex4 improves adverse pregnancy and neonatal outcomes by modestly decreasing the rate of preterm birth and drastically improving neonatal survival in a model of maternal systemic inflammation. Moreover, Ex4 treatment of dams with systemic inflammation confers protective effects on the neonates by reducing the expression and systemic concentrations of inflammatory cytokines and promoting an anti-inflammatory phenotype of neonatal immune cells. These results provide evidence that dampening maternal systemic inflammation through novel interventions such as Ex4 can improve the quality of life for neonates born to women with this clinical condition.

## Ethics Statement

All procedures were approved by the Institutional Animal Care and Use Committee (IACUC) at Wayne State University (Protocol No. A-07-03-15).

## Author Contributions

VG-F, DM, YX, BD, CV, YL, MA-H, NK, and NG-L: substantial contributions to the acquisition and/or analysis, and interpretation of data. NG-L and RR: substantial contributions to the conception, design, analysis, and interpretation of data. BP, SH, and LA-S: substantial contributions to the analysis and interpretation of data. All the authors: drafting the work or revising it critically for important intellectual content; final approval of the version to be submitted for publication; agreement to be accountable for all aspects of the work in ensuring that questions related to the accuracy and integrity of any part of the work are appropriately investigated and resolved.

## Conflict of Interest Statement

The authors declare that the research was conducted in the absence of any commercial or financial relationships that could be construed as a potential conflict of interest.
