# Supplementary material for: Inflammation-Induced Adverse Pregnancy and Neonatal Outcomes Can Be Improved by the Immunomodulatory Peptide Exendin-4
Source: Front Immunol. 2018 Jun 18;9:1291. doi: 10.3389/fimmu.2018.01291 (PMC6015905; doi:10.3389/fimmu.2018.01291)
Supplement: Supplementary file 1 [file image_1.PDF]

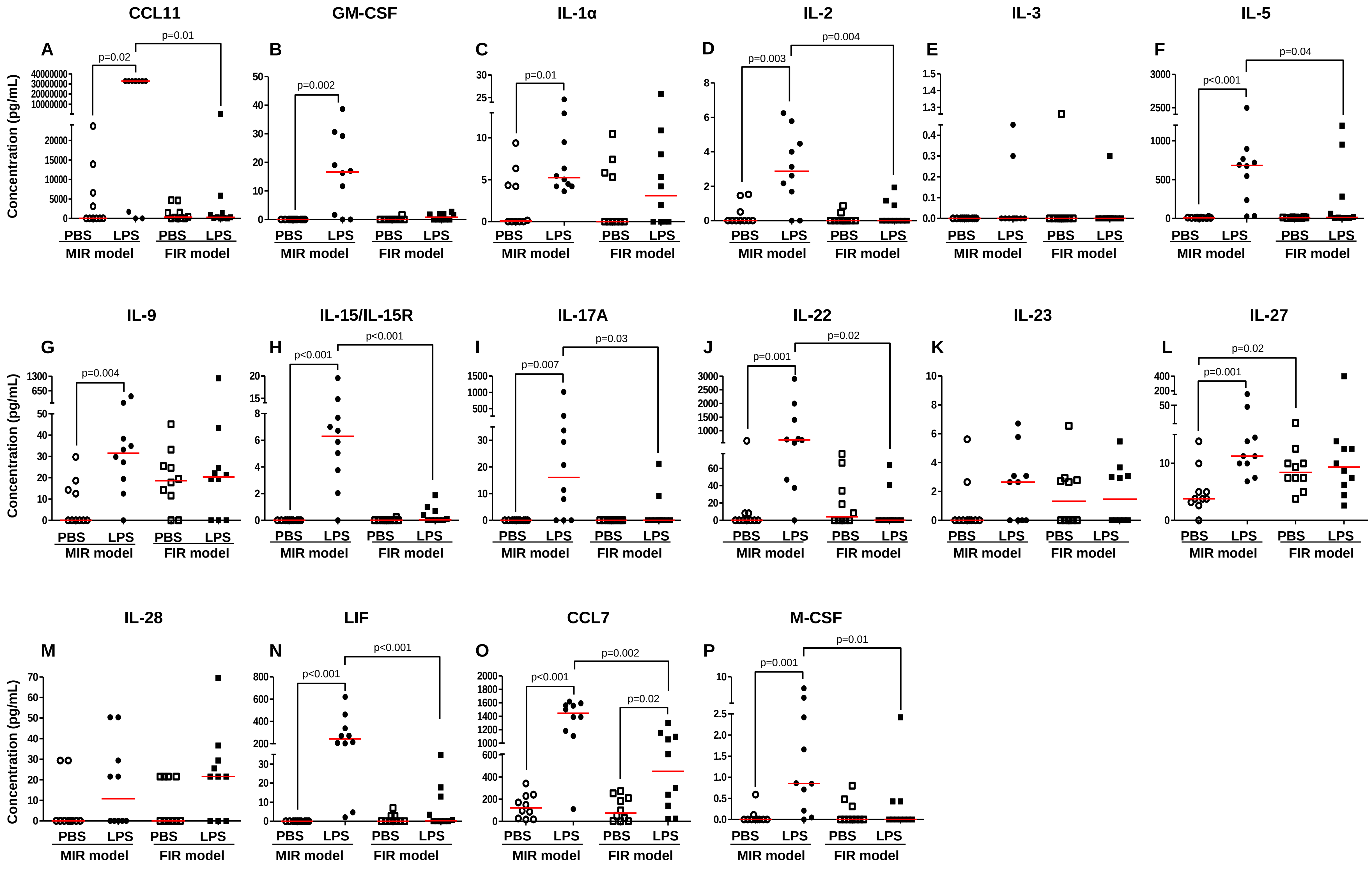

Supplementary Figure 1: Extended data for Figure 2. Concentrations of A) CCL11, B) GM-CSF, C) IL-1α, D) IL-2, E) IL-3, F) IL-5, G) IL-9, H) IL-15/IL-15R, I) IL-17A, J) IL-22, K) IL-23, L) IL-27, M) IL-28, N) LIF, O) CCL7, and P) M-CSF in the maternal serum. n=10 dams with litters per group.

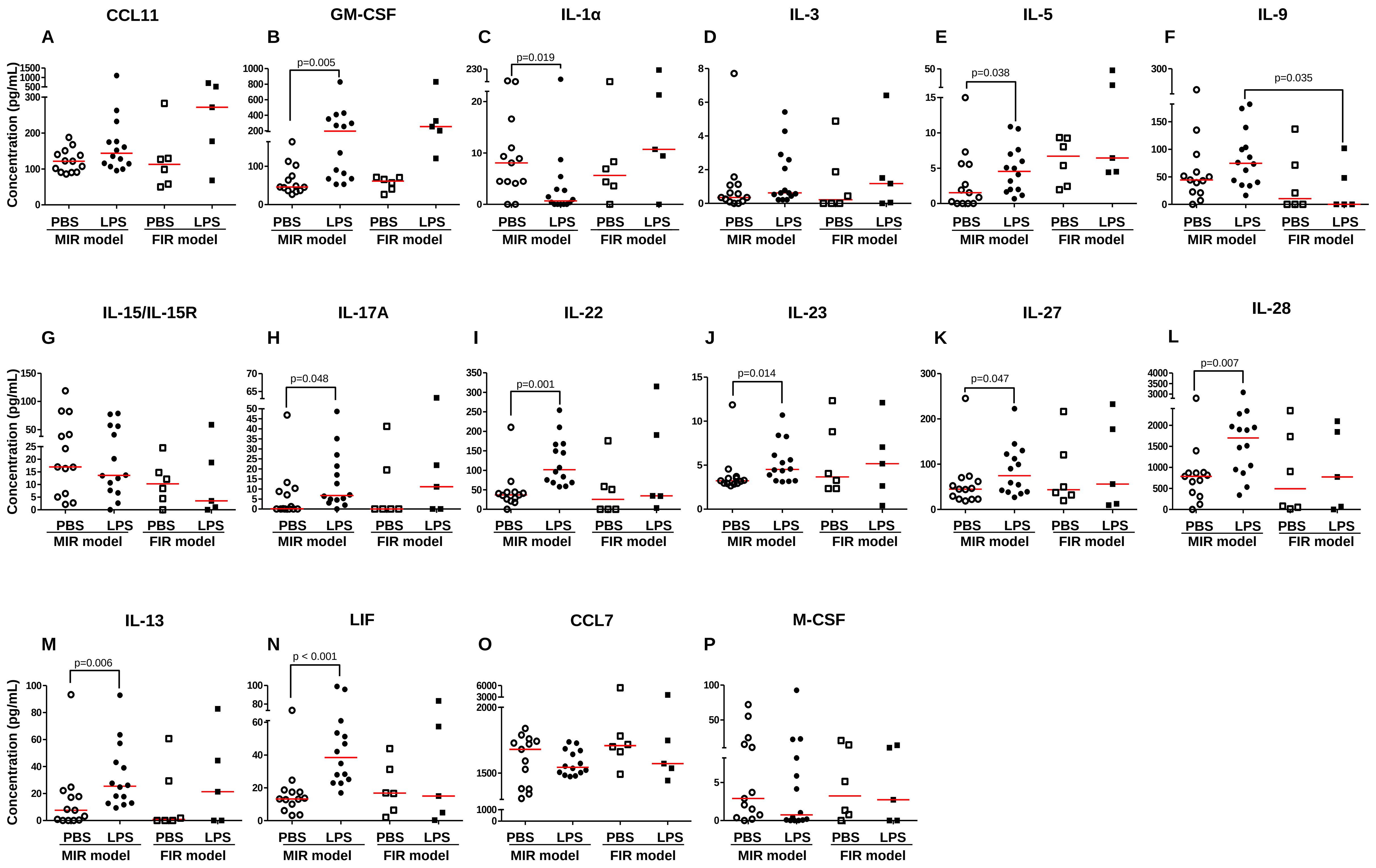

Supplementary Figure 2: Extended data for Figure 3. Concentrations of A) CCL11, B) GM-CSF, C) IL-1α, D) IL-3, E) IL-5, F) IL-9, G) IL-15/IL-15R, H) IL-17A, I) IL-22, J) IL-23, K) IL-27, L) IL-28, M) IL-13, N) LIF, O) CCL7, and P) M-CSF in the amniotic fluid. n=5-14 dams with litters per group.

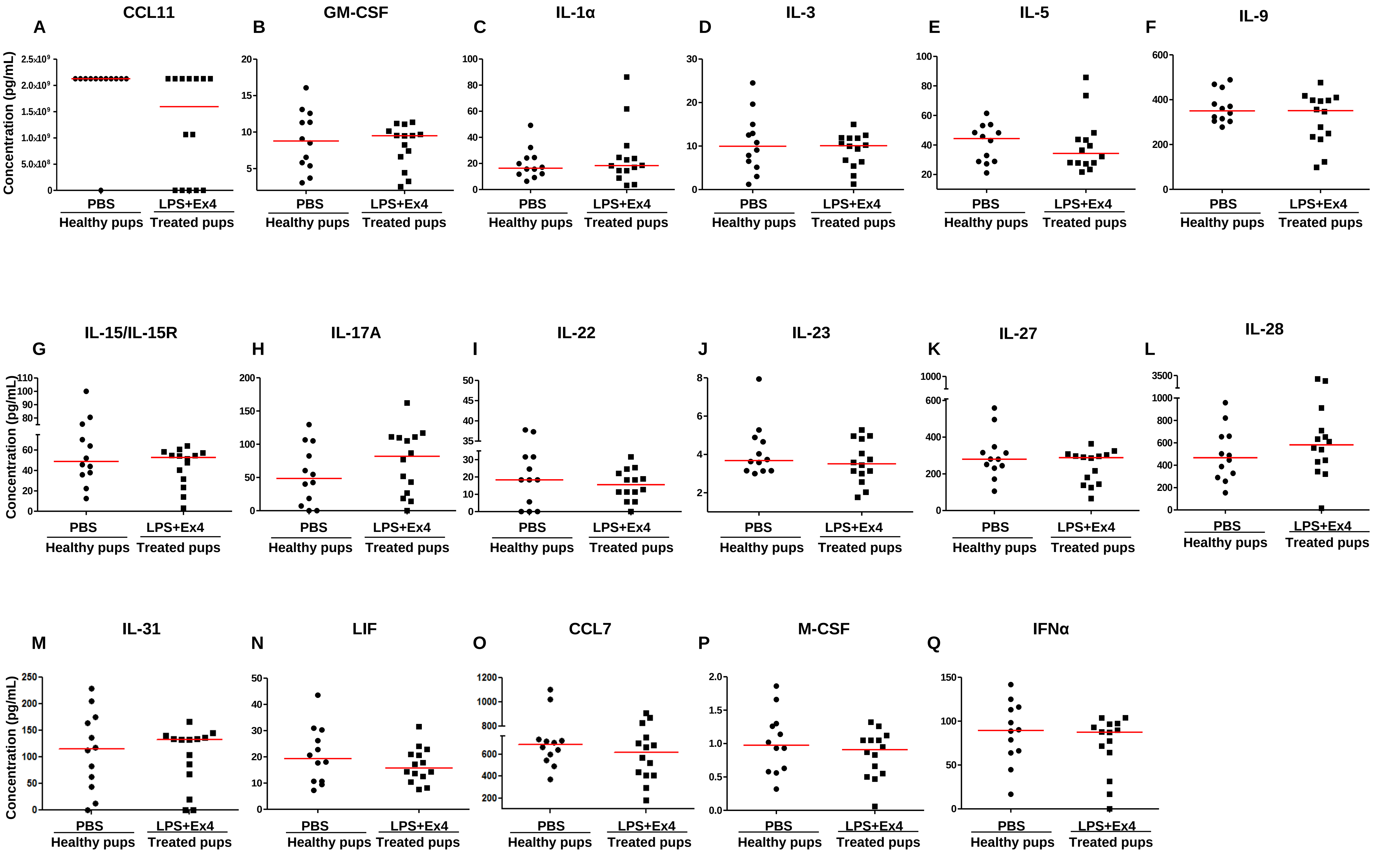

Supplementary Figure 3: Extended data for Figure 7. Concentrations of A) CCL11, B) GM-CSF, C) IL-1α, D) IL-3, E) IL-5, F) IL-9, G) IL-15/IL-15R, H) IL-17A, I) IL-22, J) IL-23, K) IL-27, L) IL-28, M) IL-31, N) LIF, O) CCL7, P) M-CSF, and Q) IFN-α in the neonatal plasma. n=12-14 neonates per group.

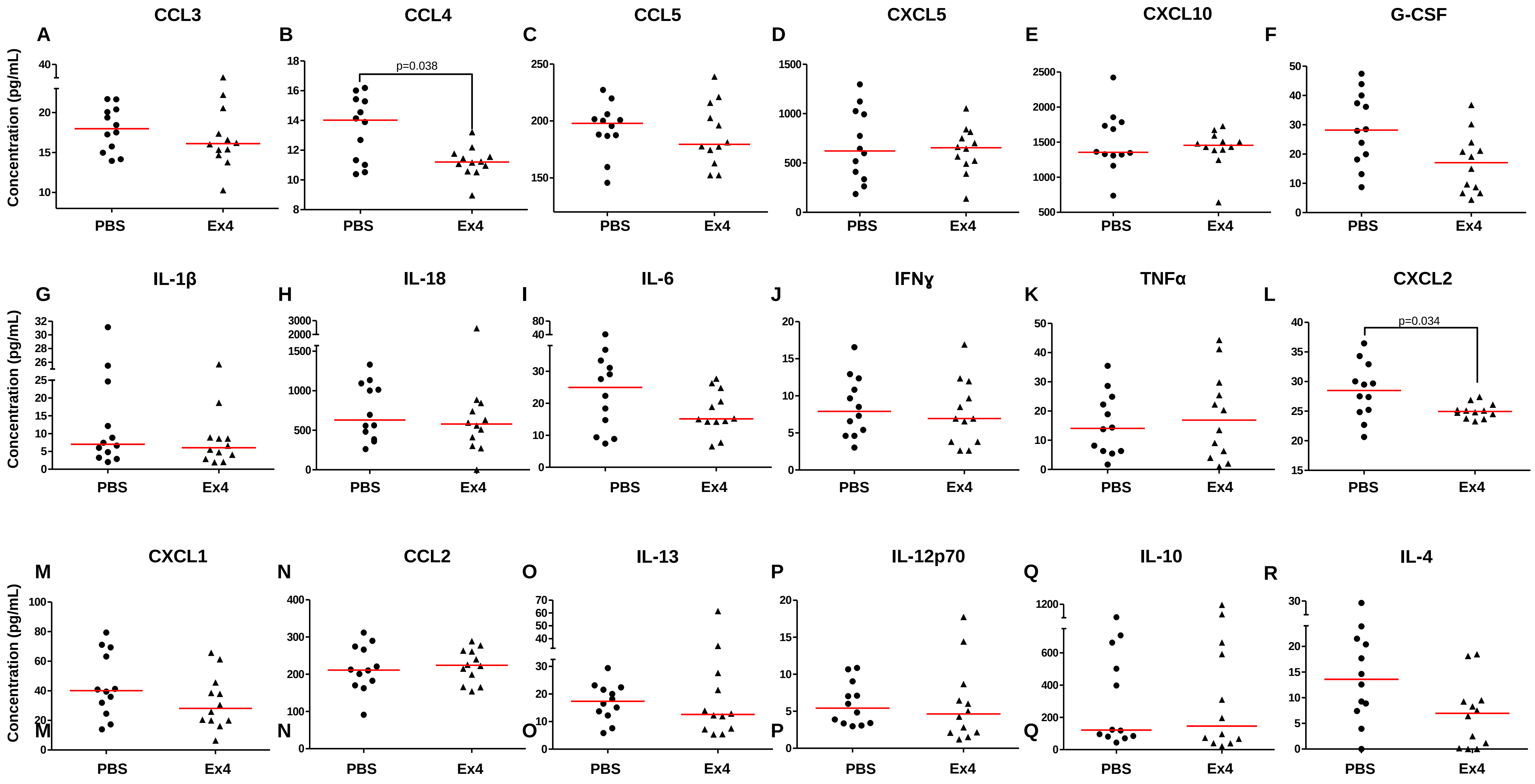

Supplementary Figure 4: Plasma cytokine concentrations from neonates born to exendin-4-treated dams compared to those from 1X phosphate-buffered saline-treated controls. Concentrations of A) CCL3, B) CCL4, C) CCL5, D) CXCL5, E) CXCL10, F) G-CSF, G) IL-1β, H) IL-18, I) IL-6, J) IFNɣ, K) TNFα, L) CXCL2, M) CXCL1, N) CCL2, O) IL-13, P) IL-12p70, Q) IL-10, and R) IL-4 in the neonatal plasma. n=12 neonates per group.

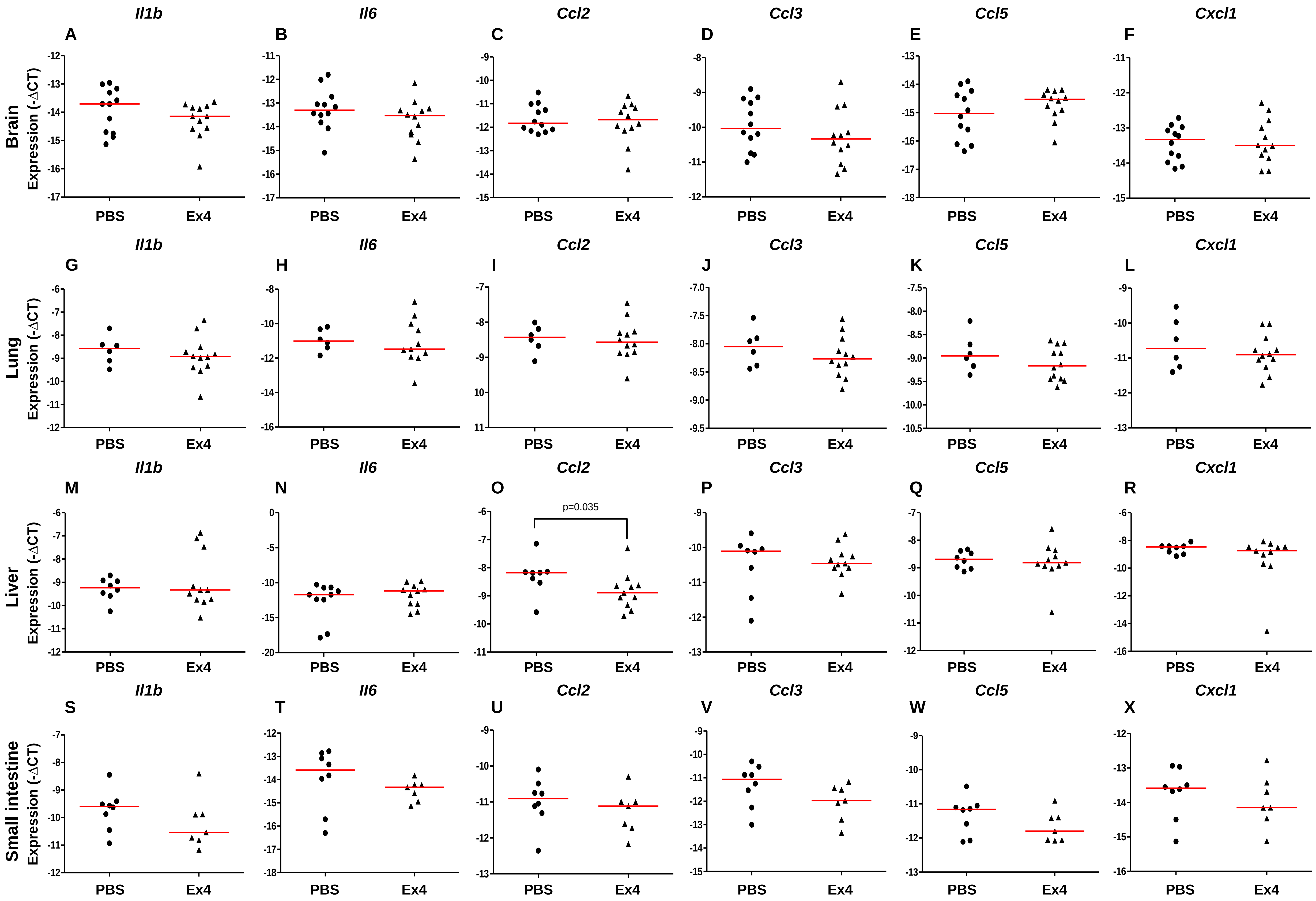

Supplementary Figure 5: Tissue cytokine expression in neonates born to exendin-4-treated dams compared to those from 1X phosphate-buffered saline-treated controls. Expression levels of *Il1B, Il6, Ccl2, Ccl3, Ccl5,* and *Cxcl1* in the neonatal brain (A-F), lung (G-L), liver (M-R), and small intestine (S-X). n=12 neonates per group.

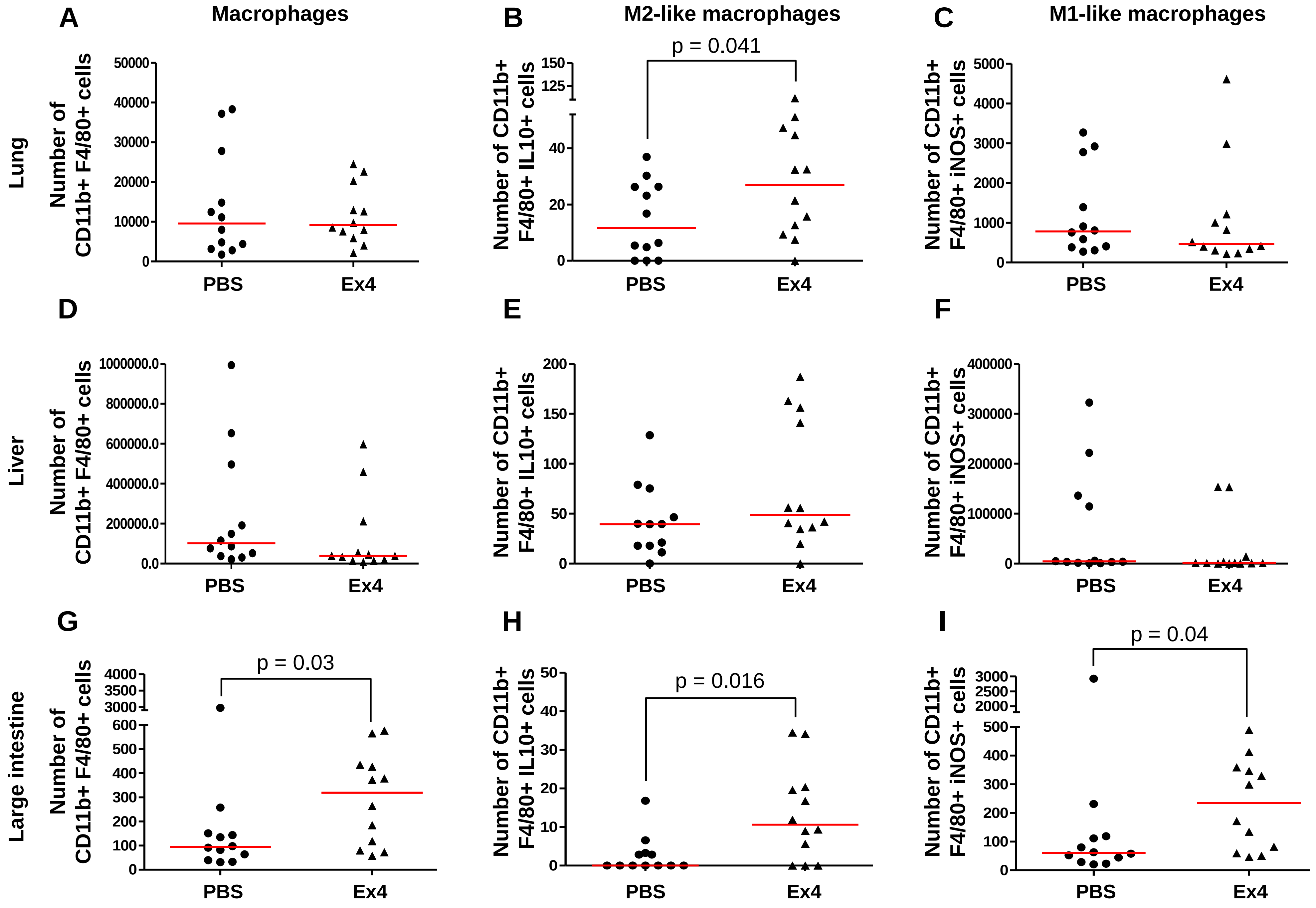

Supplementary Figure 6: Macrophages in neonates from exendin-4-treated dams compared to those from 1X phosphate-buffered saline-treated controls. Numbers of total macrophages and M1- or M2-polarized macrophages in the neonatal lung (A-C), liver (D-F), and large intestine (G-I). n=12 neonates per group.

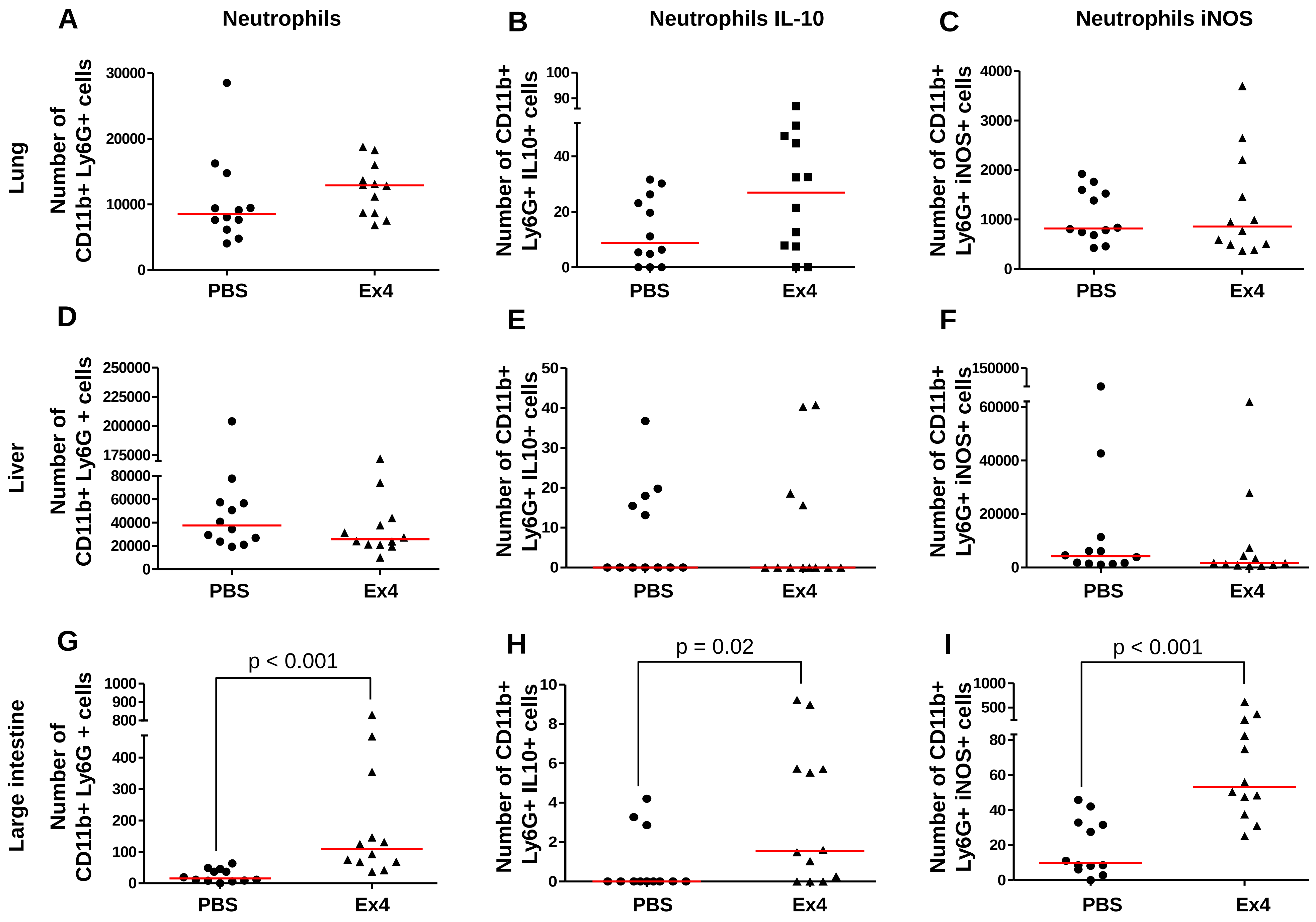

Supplementary Figure 7: Neutrophils in neonates from exendin-4-treated dams compared to those from 1X phosphate-buffered saline-treated controls. Numbers of total neutrophils and IL-10- or iNOS-expressing neutrophils in the neonatal lung (A-C), liver (D-F), and large intestine (G-I). n=12 neonates per group.

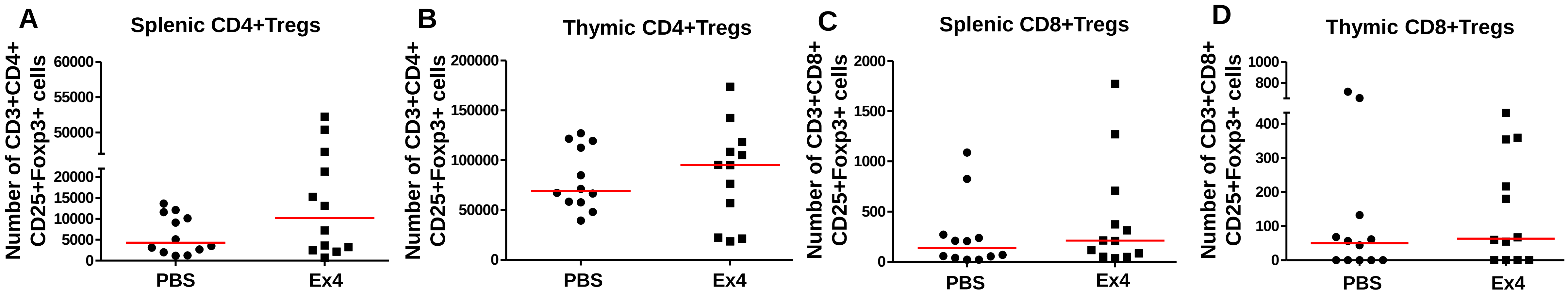

Supplementary Figure 8: Regulatory T cells in neonates from exendin-4-treated dams compared to those from 1X phosphate-buffered saline-treated controls. Numbers of CD4+ (A-B) and CD8+ (C-D) regulatory T cells in the neonatal spleen and thymus. n=12 neonates per group.
