# Supplementary material for: Inflammation-Induced Adverse Pregnancy and Neonatal Outcomes Can Be Improved by the Immunomodulatory Peptide Exendin-4
Source: Front Immunol. 2018 Jun 18;9:1291. doi: 10.3389/fimmu.2018.01291 (PMC6015905; doi:10.3389/fimmu.2018.01291)
Supplement: Supplementary file 2 [file table_1.PDF]

Supplementary Table 1. TaqMan® gene expression assays used in this study

Gene name	Gene Symbol	Assay ID
Actin, beta	*Actb*	Mm04394036_g1
Glyceraldehyde-3-phosphate dehydrogenase	*Gapdh*	Mm99999915_g1
Glucuronidase, beta	*Gusb*	Mm01197698_m1
Heat shock protein 90 alpha (cytosolic), class B member 1	*Hsp90ab1*	Mm00833431_g1
Interleukin 1 beta	*Il1b*	Mm00434228_m1
Interferon gamma	*Ifng*	Mm01168134_m1
Interleukin 6	*Il6*	Mm00446190_m1
Interleukin 18	*Il18*	Mm00434226_m1
Interleukin 23, alpha subunit p19	*Il23a*	Mm00518984_m1
Interleukin 33	*Il33*	Mm00505403_m1
Transforming growth factor, beta 1	*Tgfb1*	Mm01178820_m1
NLR family, pyrin domain containing 3	*Nlrp3*	Mm00840904_m1
Caspase 1	*Casp1*	Mm00438023_m1
SR-related CTD-associated factor 11	*Scaf11 (Casp11)*	Mm01297328_m1
Antigen identified by monoclonal antibody Ki 67	*Mki67 (Ki-67)*	Mm01278617_m1
Chemokine (C-C motif) ligand 2	*Ccl2*	Mm00441242_m1
Chemokine (C-C motif) ligand 3	*Ccl3*	Mm00441259_g1
Chemokine (C-C motif) ligand 5	*Ccl5*	Mm01302427_m1
Chemokine (C-C motif) ligand 17	*Ccl17*	Mm01244826_g1
Chemokine (C-C motif) ligand 22	*Ccl22*	Mm00436439_m1
Chemokine (C-X-C motif) ligand 1	*Cxcl1*	Mm04207460_m1
Chemokine (C-X-C motif) ligand 10	*Cxcl10*	Mm00445235_m1
Tumor necrosis factor	*Tnf*	Mm00443258_m1
Selectin, lymphocyte	*Sell (L-selectin)*	Mm00441291_m1
Intercellular adhesion molecule 1	*Icam1*	Mm00516023_m1
Intercellular adhesion molecule 2	*Icam2*	Mm00494862_m1
Vascular cell adhesion molecule 1	*Vcam1*	Mm01320970_m1
CD3 antigen, epsilon polypeptide	*Cd3e*	Mm01179194_m1
Nucleotide-binding oligomerization domain containing 1	*Nod1*	Mm00805062_m1
Nucleotide-binding oligomerization domain containing 2	*Nod2*	Mm00467543_m1
High mobility group box 1	*Hmgb1*	Mm00849805_gH

NLR family, CARD domain containing 4	*Nlrc4*	Mm01233151_m1
NLR family, pyrin domain containing 1A	*Nlrp1a*	Mm03047263_m1
Absent in melanoma 2	*Aim2*	Mm01295719_m1
PYD and CARD domain containing	*Pycard*	Mm00445747_g1
