# Supplementary material for: Inflammation-Induced Adverse Pregnancy and Neonatal Outcomes Can Be Improved by the Immunomodulatory Peptide Exendin-4
Source: Front Immunol. 2018 Jun 18;9:1291. doi: 10.3389/fimmu.2018.01291 (PMC6015905; doi:10.3389/fimmu.2018.01291)
Supplement: Supplementary file 3 [file table_2.PDF]

Supplementary Table 2. Antibodies used for immunophenotyping.

Antigen	Symbol	Fluorophore	Clone	Company	Catalog number
CD11b	CD11b	PE-CF594	M1/70	BD Biosciences	562287
Lymphocyte antigen 6G	Ly6G	APC	1A8	BD Biosciences	560599
F4/80	F4/80	APC-eFluor 780	BM8	eBioscience	47-4801-82
CD3	CD3	FITC	145-2C11	BD Biosciences	533062
CD4	CD4	APC	RM4-5	BD Biosciences	553051
CD8	CD8	PE-CF594	53-6.7	BD Biosciences	562283
CD25	CD25	PECy7	PC61	BD Biosciences	552880
Inducible nitric oxide synthase 2	iNOS	PE	CXNFT	eBioscience	12-5920-82
Interleukin 10	IL-10	AF700	JES5-16E3	eBioscience	56-7101-82
Forkhead box p3	Foxp3	V450	MF23	BD Biosciences	561293
